# Intratibial Injection of Human Multiple Myeloma Cells in NOD/SCID IL-2Rγ(Null) Mice Mimics Human Myeloma and Serves as a Valuable Tool for the Development of Anticancer Strategies

**DOI:** 10.1371/journal.pone.0079939

**Published:** 2013-11-06

**Authors:** Julia Schueler, Dagmar Wider, Kerstin Klingner, Gabrielle M. Siegers, Annette M. May, Ralph Wäsch, Heinz-Herbert Fiebig, Monika Engelhardt

**Affiliations:** 1 Department of Hematology and Oncology, University of Freiburg Medical Center, Freiburg, Germany; 2 Department for Invivo Tumorbiology, Oncotest, Freiburg, Germany; 3 Department of Anatomy & Cell Biology, Schulich School of Medicine, Western University, London, Ontario, Canada; 4 Department of Pathology, University of Freiburg Medical Center, Freiburg, Germany; French Blood Institute, France

## Abstract

**Background:**

We systematically analyzed multiple myeloma (MM) cell lines and patient bone marrow cells for their engraftment capacity in immunodeficient mice and validated the response of the resulting xenografts to antimyeloma agents.

**Design and Methods:**

Using flow cytometry and near infrared fluorescence in-vivo-imaging, growth kinetics of MM cell lines L363 and RPMI8226 and patient bone marrow cells were investigated with use of a murine subcutaneous bone implant, intratibial and intravenous approach in NOD/SCID, NOD/SCID treated with CD122 antibody and NOD/SCID IL-2Rγ(null) mice (NSG).

**Results:**

Myeloma growth was significantly increased in the absence of natural killer cell activity (NSG or αCD122-treated NOD/SCID). Comparison of NSG and αCD122-treated NOD/SCID revealed enhanced growth kinetics in the former, especially with respect to metastatic tumor sites which were exclusively observed therein. In NSG, MM cells were more tumorigenic when injected intratibially than intravenously. In NOD/SCID in contrast, the use of juvenile long bone implants was superior to intratibial or intravenous cancer cell injection. Using the intratibial NSG model, mice developed typical disease symptoms exclusively when implanted with human MM cell lines or patient-derived bone marrow cells, but not with healthy bone marrow cells nor in mock-injected animals. Bortezomib and dexamethasone delayed myeloma progression in L363- as well as patient-derived MM cell bearing NSG. Antitumor activity could be quantified via flow cytometry and in vivo imaging analyses.

**Conclusions:**

Our results suggest that the intratibial NSG MM model mimics the clinical situation of the disseminated disease and serves as a valuable tool in the development of novel anticancer strategies.

## Introduction

Multiple myeloma (MM) is characterized by monoclonal plasma cell proliferation, where the latter have undergone somatic hypermutation, antigen selection and IgH switching in germinal centers. Clinical features of the disease are excessive production of monoclonal immunoglobulin, renal impairment, hyperviscosity, bone pain, pathologic fractures and anemia due to plasma cell infiltration of bone and bone marrow (BM) spaces [1,2].

Appropriate animal models for hematological malignancies are highly attractive, because they allow the study of the biology and underlying disease mechanisms. They also constitute a major prerequisite for rapid bench-to-bedside translation of investigational anticancer therapies. Nevertheless, it has been challenging to establish predictive models using MM cell lines or primary patient material, and even more demanding to simulate the natural milieu, where MM takes place [3,4]. Human tumor xenograft models using immunodeficient mice mimic the clinical situation [5,6], however, models involving subcutaneous or intraperitoneal tumor implantation do not accurately reproduce the growth behavior and drug sensitivity patterns of leukemia or lymphoma diseases. In particular, they do not reflect the systemic nature of diffuse myeloma lesions involving the BM microenvironment, which plays a pivotal role in MM. Apart from SCID-hu and SCID-synth-hu models [7], NOD/SCID IL-2Rγ(null) mice (NSG) have been reported to be better recipients for xenotransplantations, because of improved engraftment associated with abolishment of residual immune function and lack of thymic lymphoma development accompanied by an extended lifespan [7,8]. 

Here, we studied the orthotopic engraftment of L363 and RPMI8226 as well as of MM patient-derived BM cells under different growth conditions. We systematically analyzed, whether the lack of natural killer (NK) cell activity, either with use of a NK-depleting anti-mouse-CD122-antibody or by lack of signaling through the common γ-chain in NSG, influenced myeloma growth *in vivo*. Growth kinetics of L363 and RPMI8226 were investigated with use of a murine bone implant (b.i.), intravenous (i.v.) or intratibial (i.t.) approach in NOD/SCID as compared to NSG. We further validated the i.t. NSG approach using MM patient-derived BM cells. Beyond that, we performed antitumor activity tests using dexamethasone and bortezomib in the human L363- and MM patient-derived model proving the capability of the model to assess clinical tumor response. We monitored tumor growth and antitumor activity by flow cytometry and whole-body fluorescence-based in-vivo-imaging (IVI) using dyes in the near infra-red range. Since these dyes are characterized by a deep tissue penetration and minimal autofluorescence, they provide an excellent signal to noise ratio and are thus a highly sensitive tool for myeloma disease detection [4,9]. Our analyses demonstrate the successful engraftment of two cell lines in two different mouse strains using various routes of transplantation and of tumor samples of 11 patients with symptomatic MM when engrafted i.t. in NSG. Albeit our model may resembles more advanced myeloma disease, since systemic disease sites like spleen, blood and liver were detected therein, which has similarly been described by others [4,10,11], this does not reduce the suitability and technical merits of our model for innovative drug testing, because our goal to define best murine conditions with established cell lines and patient-derived BM cells was solved therein. We show here that 1) the NSG i.t. model is superior over the NOD/SCID i.t. model for outgrowth of cancer cells, 2) fluorescence-based IVI can be used for analyzing growth of MM cells in vivo, 3) L363 cells engraft better than RPMI8226 cells and 4) MM BM cells can engraft mice using the NSG i.t. model and can be used to assess and personalize treatment strategies.

## Design and Methods

### In vitro analyses

#### Cell culture conditions

The human multiple myeloma cell line (MMCL) RPMI8226 and human plasma cell leukemia cell line L363 were obtained from the DSMZ (Braunschweig, Germany). All cells were grown in RPMI 1640 medium supplemented with 10% fetal bovine-serum, 1% penicillin (100U/mL), streptomycin (100U/mL) and 1% L-glutamine (all from GIBCO-BRL, Grand Island, NY). Cells were maintained at 37°C in 5% CO_2_ / 95% air atmosphere and split and seeded in fresh medium every three (L363) and six (RPMI8226) days. The immunophenotype was determined using HLA-A,B,C-FITC, CD138-PE, CD45-FITC, HLADR-PE, CXCR4-FITC and CD38-PE conjugated mouse anti-human-IgG1 (BD Pharmingen, Germany). Cells (1x10^6^) were incubated with the primary antibody or isotype control and the mean fluorescence intensity analyzed by flow cytometry. The tumor load was assessed by measurement of the percentages of human HLA-A,B,C, CD138, CD45, HLA-DR and CD38 positive cells after red blood cell lysis with ammonium chloride. All samples were analyzed on a FACSort (Becton Dickinson) flow cytometer which recorded 50,000 events. Staining was performed in the presence of CD16/CD32 Abs to block non-specific staining (rat anti-mouse CD16/CD32/FcγIII/II receptor IgG2b, BD Pharmingen).

### *In vivo* models of human MM

#### Mice

NOD.Cg-Prkdc^scid^-mice (NOD/SCID) were obtained from Taconic, Denmark, and non-obese diabetic severe combined immunodeficient mice with a deficient interleukin-2 receptor gamma chain (NSG) from Jackson Laboratory, Bar Harbor, USA under microisolators in barrier conditions. At 6-8 weeks of age, mice were injected with L363, RPMI8226 or MM patient-derived cells. Engraftment in different mouse strains 35 days after cell injection was assessed by means of take-rates (= number of tumor-bearing mice) and quantitative MM cell engraftment via flow-cytometry and fluorescence-based-*in vivo* imaging (IVI) in different organs. As e.g. L363 cells do not secrete immunoglobulins, the determination of tumor load via serum heavy or light chain markers produced by MM cells was not performed, nevertheless as sensitive methods for monitoring tumor load take-rates, quantitative *h*HLA-ABC or *h*CD138 expression by flow cytometry and fluorescence-IVI were performed as well as monitoring of disease symptoms (lethargy, hunched posture, failure to eat and drink). After mice had been sacrificed, tissue from the injection site, BM from femurs, organs (spleen and liver) and peripheral blood (PB) were collected for phenotypic analysis. Engrafted mice were sacrificed when they developed disease symptoms. Some mice were sacrificed earlier (28-29 days after implantation) to monitor disease progression. All surgery was performed under isoflurane anaesthesia, and all efforts were made to minimize suffering. 

#### Subcutaneous injection into bone implants (b.i.), intravenous (i.v.) and intratibial (i.t.) cell injection

A total of 60 mice received subcutaneous implants of juvenile murine long bone. Femura of 4-6 week old NOD/SCID mice were prepared, all soft tissue removed and partly opened at one end. The bone cavity was flushed with PBS. After that, the hollow bones were filled with 2x10^5^ L363 or RPMI8226 cells resuspended in PBS prior to implantation into mice. In such a way prepared bones were implanted subcutaneously into recipient mice. Moreover, 44 mice were i.v. inoculated with 2x10^5^ L363 or RPMI8226 cells, and 51 mice with 2x10^5^ L363 or RPMI8226 cells by direct i.t.-injection. For this i.t.-injection, mice were briefly anesthetized with isoflurane. Initial experiments used dye to confirm accuracy of the injection. The suitability of i.t.-implanted NSG was further pursued with use of samples from 11 consecutive MM patients (2x10^5^ - 2x10^6^ mononuclear cells/mouse). Control mice were inoculated with healthy donor BM cells (2x10^5^ mononuclear cells/mouse) or PBS alone (mock-injection). 

#### Pre-treatment with anti-mouse-CD122-antibody

In order to assess, whether pre-treatment of the CD122-antibody enhanced engraftment in NOD/SCID, a total of 42 mice received a single dose of 250µg/mouse α-mouse-CD122-antibody (PharMingen, Europe) via intraperitoneal injection 24hrs prior to L363- or RPMI8226-injection. This pre-treatment was used to block the activity of mouse NK cells via IL-2 receptor beta chain, thereby potentially enhancing cancer cell engraftment.

#### In vivo imaging (IVI)

IVI was performed on patient-derived MM bearing mice 38, 46 and 56 days post injection with the Kodak Image Station in vivoFX using 0.02mg *h*HLA-A,B,C (day 38), *h*CD45 (day 46) and *h*CD138 antibodies (day 56) labelled with Alexa750 dye (BeckmannCoulter, Germany). IVI was performed up to once weekly using the labelled *h*CD138 antibody. All three antibodies were used to assess which was most sensitive to determine human L363, RPMI8226 and patient-derived cell engraftment. For optimal localization of the fluorescent region, animals were x-rayed and the two pictures merged. After antibody injection, mice were anesthetized by isoflurane inhalation and images were taken with a Kodak *in vivo* imaging system (Kodak Image Station *in vivo* FX). To ensure that tagged-antibody application did not interfere with mouse tissue or applied therapeutics, non-tumor bearing mice were injected with the same dose of tagged antibodies as tumor bearing mice and fluorescence intensity was determined which did not depict unspecific binding. Specificity of the fluorophore coupled antibodies was determined *in vitro* via cell binding assay (Figure S1). To determine residual net intensity (=background) in animals pre-treated with different antibodies, reinjection of the three different antibodies coupled to the same fluorochrome was performed in the same chronological order and time course as in the main analysis and net intensity was compared before and after injection of the antibody (Figure S2+S3) The detection limit of the CCD camera was determined in vitro via cell binding assay as well as in vivo (Figure S4). The selected surface antigens *h*HLA-A,B,C, *h*CD138 and *h*CD45 were expressed on both cell lines and patient-derived MM cells (Figure S5). 

#### Treatment of tumor-bearing mice with different anti-MM agents

In a first experiment, a total of 24 NSG received i.t.-injections of 2x10^5^ L363 cells: seven days thereafter, 18 mice were randomized into three groups of six animals each, treated with aqua (10ml/kg/d intraperitoneally [i.p.] on days 7-11 and 14-18), dexamethasone (3mg/kg/d i.p. on days 7-11 and 14-18; Jenapharm) or bortezomib (0.7mg/kg/d i.v. on days 7, 11 and 18; Janssen). Six additional mice were analysed on the first treatment day (d7) to determine tumor load before the respective therapies. MM cell engraftment was determined by flow cytometry.

In order to assess, whether tumor response was equally well detected with fluorescence-based-IVI, tumor growth and repression was monitored via IVI using *h*CD138 antibody in a second experiment: 12 NSG received i.t.-injections of 2x10^5^ L363 cells and each six mice again received either aqua or bortezomib. Cell engraftment was assessed in the BM and distant organs (spleen and liver). In a third experiment, 2x10^6^ patient derived MM cells per mouse were i.t.-injected in 30 NSG. Tumor growth was monitored via IVI using *h*CD138 antibody and flow cytometry on BM, PB and spleen. Eleven days after tumor cell injection, animals were stratified into three groups of 10 mice each and treatment with dexamethasone (3mg/kg/d i.p. on days 11-15 and 25-29; Jenapharm), bortezomib (0.7mg/kg/d i.v. on days 11, 15, 19 and 22; Janssen) or control vehicle (10ml/kg/d i.p., on days 11-15 and 25-29) was begun. Myeloma growth repression was calculated as the reduction of human tumor cells compared to untreated mice. 38 days after tumor cell inoculation, animals were sacrificed and tumor load determined via flow cytometry and immunohistochemistry.

### Histological and immunohistochemical analysis

Murine long bones were fixed in 4% formaldehyde and decalcified in a mixture of EDTA disodium salt and tris-(hydroxymethyl)aminomethane (THAM; TRIZMA, Sigma-Aldrich, Munich, Germany) at pH7. Afterwards the tissue was processed and paraffin embedded. For conventional light microscopy 3μm thick serial sections were deparaffinized in xylene and graded alcohols and stained with haematoxylin eosin (HE). Immunohistochemistry was performed with the Autostainer Link 48 (DAKO) using antibodies against CD138 (Flex monoclonal mouse anti-human, Clone MI15, RTU, DAKO) and Kappa (Flex polyclonal rabbit anti-human Kappa light chains, RTU, DAKO). The slides were deparaffinized and pretreated in the PT Link module (DAKO) for 15 minutes, using EnVision Flex Target Retrieval Solution, low pH (50 x concentrate, DAKO, K8005), followed by a peroxidase block (DAKO, SM801) for 5 (CD138) or 10 (Kappa) minutes. After 5 (Kappa)/10 (CD138) minutes of incubation with the primary antibodies the signal for CD138 was enhanced by 15 minutes of incubation with Flex+ Mouse (Linker) (DAKO, K8021). Visualisation was performed using the EnVision Flex detection system (DAKO, K8000) according to the manufacturer’s instructions. The sections were counterstained with haematoxylin and subsequently mounted. The histologic images were obtained with an Zeiss Axioskop microscope with Plan Neofluar (20x/0,50 or 40x/0,75) objectives, equipped with a digital camera (LC50; Olympus soft imaging solutions) using LCmicro software, Version 5.2 (Olympus soft imaging solutions). The brightness was adjusted, applying Adobe Photoshop CS2 Version 9.0.

### Ethics statement

This study was carried out in strict accordance with the recommendations in the Guide for the Care and Use of Laboratory Animals of the Society of Laboratory Animals (GV SOLAS). All animal experiments were approved by the Committee on the Ethics of Animal Experiments of the regional council (Regierungspräsidium Freiburg, Abt. Landwirtschaft, Ländlicher Raum, Veterinär- und Lebensmittelwesen - Ref. 35, permit-G-09/59). The analyses were performed according to the guidelines of the Declaration of Helsinki and good clinical practice. All patients and the healthy donor gave their written informed consent for institutional-initiated research studies and analyses of clinical outcome studies conforming to our institutional review board guidelines. All experiments were approved by the ethical commission of the Albert Ludwig University Freiburg (permit-# EK Freiburg: 345/03).

### Statistical analysis

Student's t test, two-tailed, 1way ANOVA and log rank test were used to calculate all reported p-values. Descriptive analyses were assessed whenever appropriate and graphs (Kaplan Meier plots) were obtained using GraphPrism software (www.graphpad.com).

## Results

### Growth kinetics of L363 and RPMI8226 cells: engraftment capacity in various immunodeficient mice via subcutaneous bone implant (b.i.), intratibial (i.t.) or intravenous (i.v.) injection.

**Figure 1:**
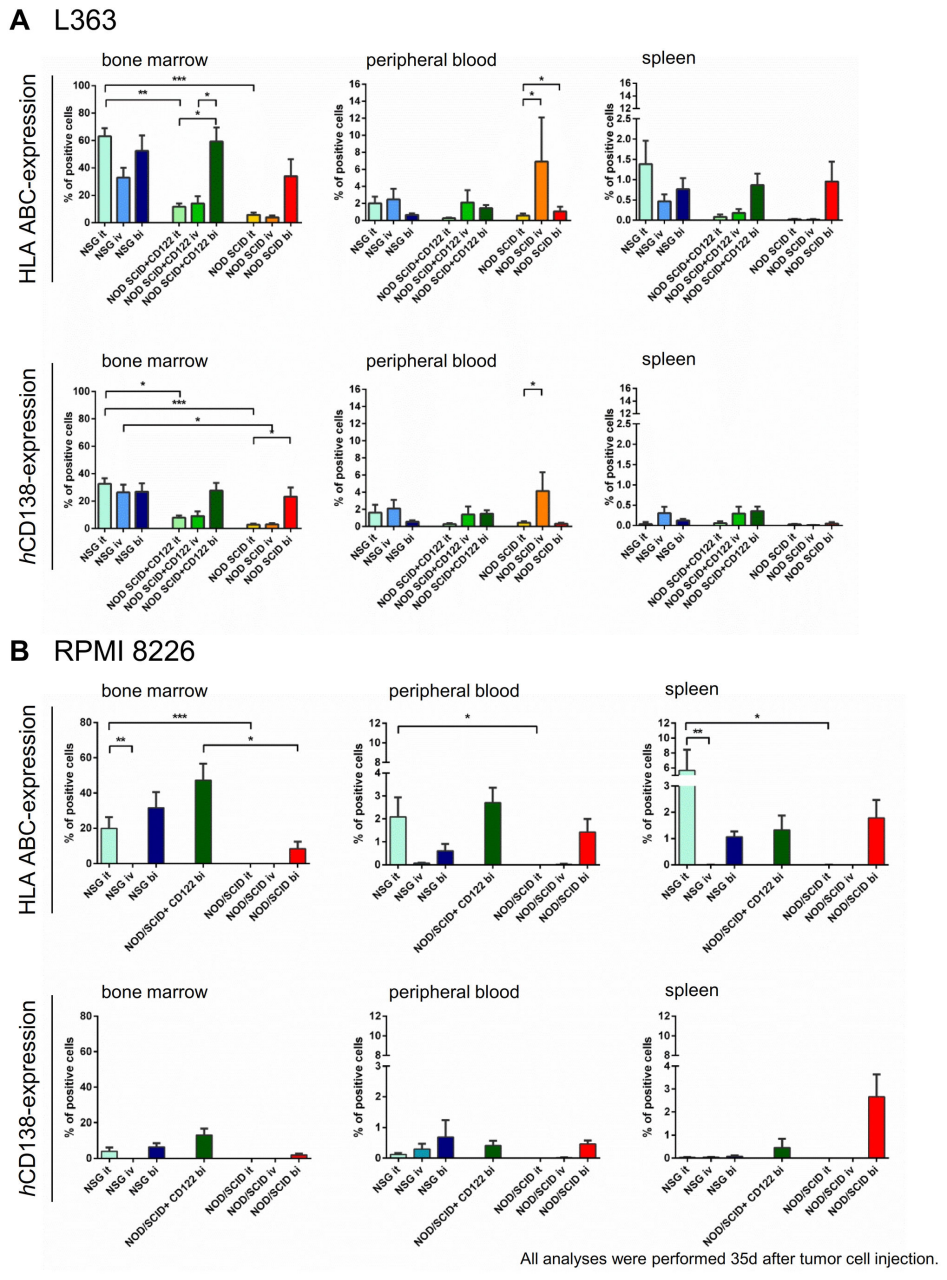
Engraftment of human L363- and human RPMI8226- cells *in vivo* determined via flow cytometry. A total of 106 mice received human L363 cells and 49 mice received human RPMI8226 cells via intratibial injection (it, L363: n=36, RPMI8226: n=15), intravenous injection (iv, L363: n=34, RPMI8226: n=10) or subcutaneous long bone implants (bi, L363: n=36, RPMI8226: n=24). Analyses were performed 35 days after tumor cell injection or when mice showed bad overall condition. Lack of NK cell activity, either by use of NSG mice or by use of *m*CD122-Ab treatment in NOD/SCID mice, and direct contact of implanted tumor cells with the murine bone marrow (BM), after it- or bi-injection, enhanced the engraftment of human MM cells. Human (*h*)HLA-A,B,C- as compared to *h*CD138-expression showed higher human tumor cell engraftment. This engraftment was most substantial in the BM, whereas peripheral blood (PB) and spleen engraftment was lower, albeit with similar patterns as in the BM. **A. Mean tumor infiltration rates of human L363- cells *invivo* determined via flow cytometry.** In the BM, high human engraftment was obtained in NSG mice, irrespective of the injection modus. This could only be obtained in NOD/SCID mice, if pretreatment with *m*CD122-Ab and bi-implantation were performed. The PB engraftment after iv-injection of human L363 cells in NOD/SCID mice was most likely induced due to the plasma cell leukemia nature of these cells [28,29]. **B. Mean tumor infiltration rates of human RPMI8226- cells *invivo* determined via flow cytometry.** Human RPMI8226 cells displayed similar engraftment patterns compared to L363 cells, albeit to a lower extend and highest BM engraftment potential with bi-injection regardless of the mouse strain. Differences between various conditions did not reach significance for all subgroups due to restricted group sizes.

**Figure 2:**
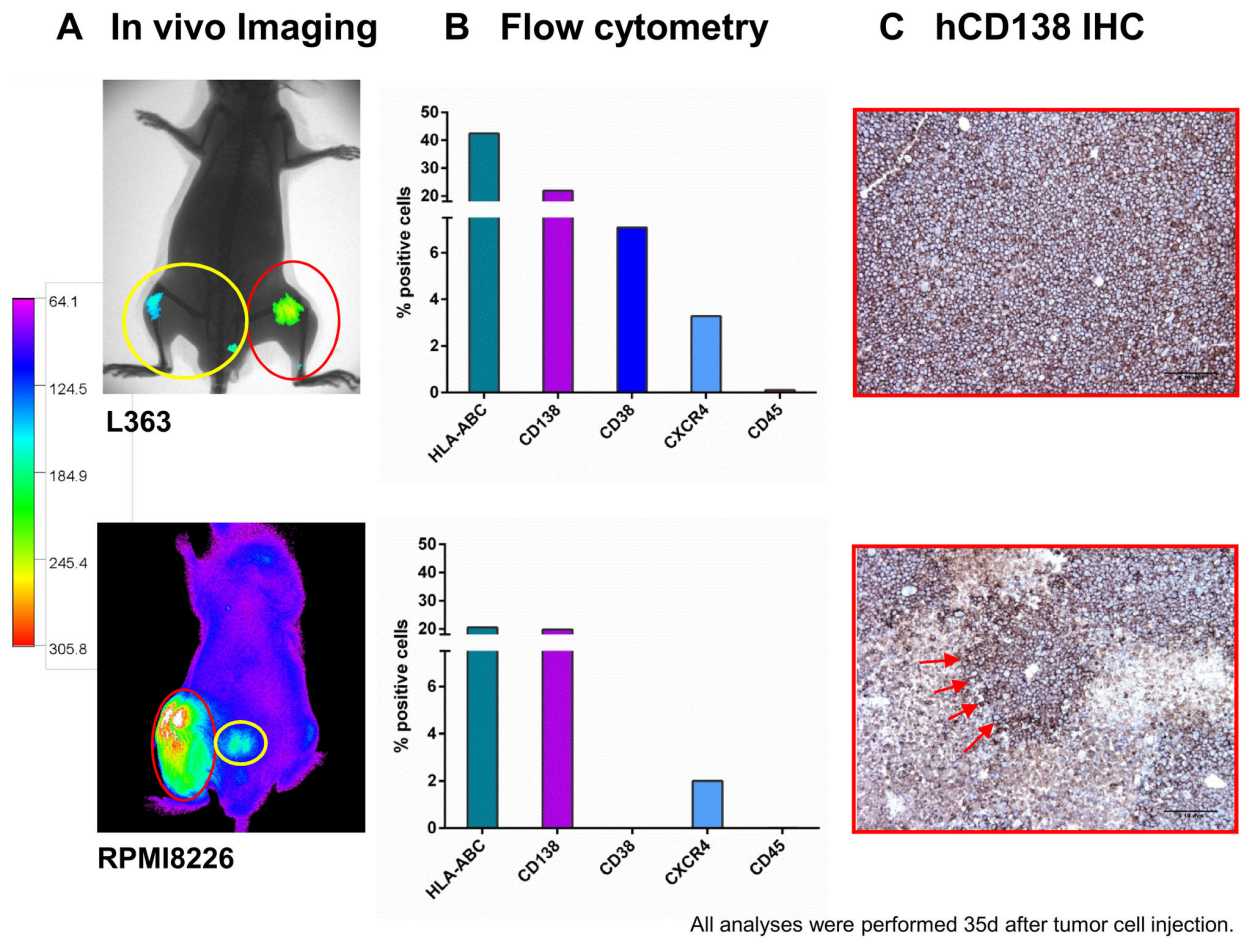
A. Detection of human L363 and RPMI8226 cells in immunodeficient mice using *h*CD138 antibody labelled with Alexa750. 20 mice per cell line were engrafted with L363 or RPMI8226 cells via different application routes. Images were taken once weekly for five weeks after implantation of the respective MM cell line with the Kodak Image Station *in* *vivo*FX. In addition to the determination of tumor load via CCD camera, animals were x-rayed and the two pictures merged for optimal localization of the fluorescent region. No x-ray was performed for the animal bearing RPMI8226 in this figure. Intraosseal tumor growth was clearly detectable by the in vivo imaging (IVI) system (red circles). Additionally, BM metastases became apparent within the adjacent tibia and lumbar spine (yellow circles). **B. Detection of human L363 and RPMI8226 cells in immunodeficient mice using flow cytometry.** IVI data for L363 and RPMI8226 were confirmed by flow cytometry 35 days after tumor cell injection showing 42% and 22% human HLA-ABC and CD138 positive cells in L363 and 21% and 20% for RPMI8226, respectively. Analyses were performed on metastatic BM lesions. **C. Detection of human L363 and RPMI8226 cells in immunodeficient mice using immunohistochemistry.** IVI data for L363 and RPMI 8226 cells were confirmed by immunohistochemistry specific for human CD138+ cells (CD138+-infiltrates, red arrows) Analyses were performed on metastatic BM lesions.

**Figure 3:**
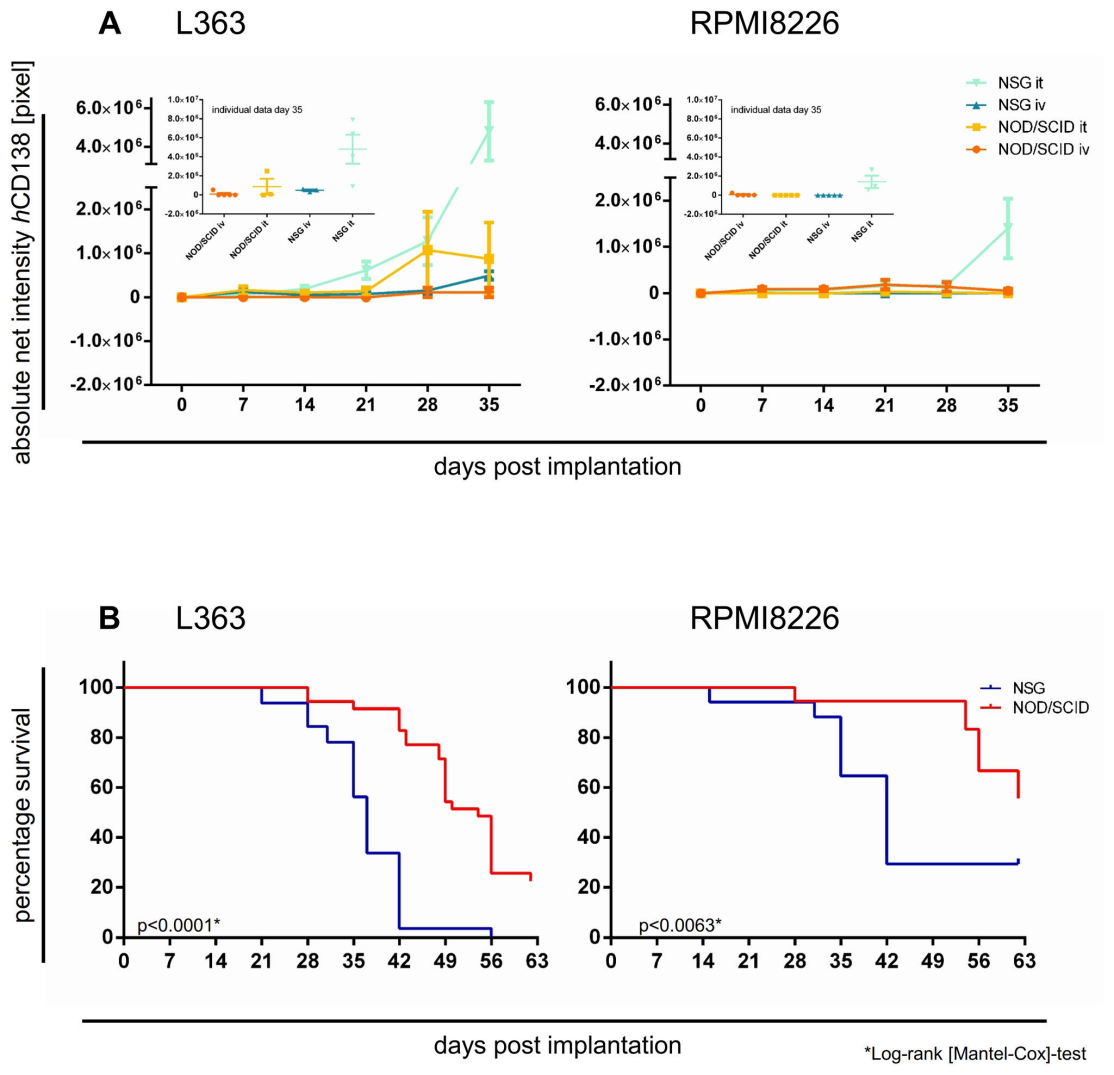
A. Detection of human L363 and RPMI8226 cells in immunodeficient mice using *h*CD138 antibody labelled with Alexa750. 20 mice per cell line were engrafted with L363 or RPMI8226 via two different application routes (it and iv). Images were taken once weekly for five weeks after implantation of the respective MM cell line with the Kodak Image Station *in* *vivo*FX. Absolute net intensity (= pixel number within the region of interest) was markedly higher after L363 than RPMI8226 injection irrespective of the route of injection and genetic background of the recipient mouse (p<0.001). Moreover, NSG permitted higher engraftment than NOD/SCID irrespective of the injected cell line (p<0.002). **B. Survival of NSG vs. NOD/SCID mice implanted with either L363 or RPMI8226 cells.** 20 mice per cell line were engrafted with L363 or RPMI8226 via two different application routes (it or iv) in NOD/SCID or NSG mice (= 40 mice in total). Survival times of tumor bearing animals were monitored as an indicator of tumor burden. Survival of NSG compared to NOD/SCID after L363 or RPMI8226 injection was substantially shorter: the impact of the genetic background of the host was statistically significant for L363 (p<0.0001, Log-rank [Mantel-Cox]-test) as well as for RPMI8226 (p<0.0063*, Log-rank [Mantel-Cox]-test).

A total of 155 mice received L363 or RPMI8226 cells via subcutaneous long bone implants (n= 60), i.t.- (n= 51) or i.v.-injection (n= 44). Several mice in each group had to be sacrificed before day 35 due to paralysis and general deterioration; the remaining mice were observed until tumor burden necessitated their sacrifice. The latest analyses were performed 62 days post-implantation. Remarkable engraftment differences were observed in different mouse strains. This was assessed by means of a) take-rates (= number of tumor-bearing mice), b) quantitative L363 / RPMI8226 engraftment via flow-cytometry (Figure 1) and fluorescence-IVI (Figure 2 +3A) and c) survival rates (Figure 3B).

Most apparent take-rate-differences were observed between NSG (i.t.) and NOD/SCID mice (i.v.; p<0.005, t-test): when L363 and RPIM8226 cells were i.t.-injected, all NSG engrafted (12/12 with L363 and 5/5 with RPMI), whereas with i.v.-injection in NOD/SCID, only 5/10 with L363 and 1/5 with RPMI8226 developed disease symptoms (Table S1).

As depicted in Figure 1A+B, *h*HLA-A,B,C- as compared to *h*CD138-positive cells were higher in all investigated compartments and settings. On both cell lines, the expression pattern of the investigated surface markers was similar (Figure S5): *h*HLA-ABC and *h*CD138 were substantially higher expressed than *h*CD45, decreasing in vivo, however, remaining well detectable (Figure S5). Highest engraftment was obtained in the BM, whereas PB and spleen engraftment was substantially lower, but showed similar pattern (Figure 1A+B).

With use of human L363 cells (Figure 1A), highest engraftment rates with i.t.-, i.v.- and b.i.-injection was obtained in the BM of NSG mice. This could similarly be obtained in NOD/SCID mice with CD122 Ab-treatment and b.i.-injection, and to an even lesser extend in NOD/SCID with b.i.-injection.

Analysis of PB samples also showed that intravenous injection of L363 cells in NOD SCID mice generated substantial *h*HLA ABC- and *h*CD138-positive cells, which was not observed to this extent with RPMI8226 cells. This increased engraftment was induced due to the plasma cell leukemia nature of the cell line (Figure 1A).

In general, the lack of NK cell activity, either by use of NSG mice or of *m*CD122-Ab treatment in NOD/SCID, as well as by direct contact of implanted tumor cells with the murine BM (both b.i.- and i.t.-injection), enhanced the engraftment of human MM cells *in vivo* (Figure 1A+B). The influence of the NK cell block was highly significant, when comparing i.t.-injection in NSG vs. NOD/SCID (p <0.0001, one-way ANOVA). In CD122-Ab treated NOD/SCID mice, the b.i. approach led to a mean of 60% *h*HLA-A,B,C positive cells in the murine BM which was similarly high with i.t. injection into NSG mice with a mean of 63% positive cells. 

RPMI8226 engraftment as depicted in Figure 1B displayed similar patterns compared to L363, albeit this was lower than with L363 cells. This engraftment difference was significant in the BM (p <0.0001, one-way ANOVA), but reached similar levels in the PB and spleen. Of interest, RPMI8226 cells showed highest BM engraftment potential with b.i.-injection regardless of the mouse strain (Figure 1B). Differences of human RPMI8226 engraftment did not for all various conditions reach significance due to lower group sizes.

### Determination of tumor cell engraftment via fluorescence-based-IVI in L363 and RPMI8226 bearing mice

Although the b.i.-NOD/SCID+CD122-Ab approach also revealed substantial BM-engraftment (Figure 1A+B), the NSG i.t. approach showed metastatic disease spread, since MM cells engrafted the i.t.-injection and disseminated to additional BM sites, thereby recapitulating the systemic nature of the disease (Figure 2A). Thirty-five days after i.t.-MMCL injection into NSG, IVI analyses allowed the detection of human MM cells which was confirmed by flow cytometry (Figure 2B) and *h*CD138 immunohistochemistry (Figure 2C).

IVI analyses verified our former flow cytometry results: the growth behavior of the injected cell line, route of injection and functionality of murine NK cells notably influenced engraftment in the BM: absolute net intensity (=pixel number within the region of interest minus background) was markedly higher in L363- than in RPMI8226-inoculated mice (Figure 3A), irrespective of the injection route and genetic background of the recipient mouse (t-test, p <0.001). Moreover, NSG permitted more substantial engraftment compared to NOD/SCID (t-test, p <0.002; Figure 3A). The i.t.-injection of both cell lines on day 35 proved highest in NSG (t-test, p <0.05); both with L363 and RPMI cells, which was substantially higher with the i.t.- vs. i.v.-injection (Figure 3A). RPMI8226 cell engraftment was most substantial with i.t.-injection into NSG (p <0.005, 1way ANOVA; Figure 3A). Only 1/5 i.v.-injected NOD/SCID mice showed minor engraftment (<5x10^5^ pixel absolute net intensity, Figure 3A).

### Murine survival after L363 and RPMI8226 injection

Murine survival provided an indicator of tumor burden that confirmed flow cytometry and IVI results. Murine survival was influenced by the genetic background, implantation site and growth behaviour of the injected cell line. Both i.t.-L363 or i.t.-RPMI8226 injection into NSG led to shortest median survival as compared to b.i.- or i.v.-injections (Table S1). Survival of NSG compared to NOD/SCID mice after L363 and RPMI injection was substantially shorter (Figure 3B + Table S1).

### Successful implantation of MM patient-derived BM cells in NSG

As i.t.-injection into NSG induced the most efficacious human cell engraftment, this approach was used for the implantation of MM patient-derived BM cells. Table S2A+B display individual and summarized patient characteristics: all were symptomatic and showed advanced disease. The median BM infiltration rate was 50%. BM of 10 MM patients and PB of one patient with plasma cell leukemia were i.t.-injected into NSG (up to 11 mice/patient sample; n=48). 

Injection of BM cells from one healthy donor (7 mice) and mock-injected NSG (n=8) served as controls. Absence of any residual fluorescence labelling at days 46 and day 56 due to previous labelling was verified using L363-bearing NSG mice to generate a similar tumor load in all examined animals. Differences in net intensity in the BM and spleen of tumor bearing mice before and after injection of the respective antibody were statistically significant for all three antibodies (unpaired t-test), as depicted in Figure S2A-C. Moreover, the net intensity of the IVI signal was equally low in all animals prior to treatment with the labelled antibody used for the latest image analysis (Figure S2B+C). The residual net intensity (=background) in animals pre-treated with different antibodies was equal when compared amongst one another as well as when compared to animals that had never received fluorochrome-coupled antibody treatment, both within the BM and spleen (one way ANOVA; Figure S3).

**Figure 4:**
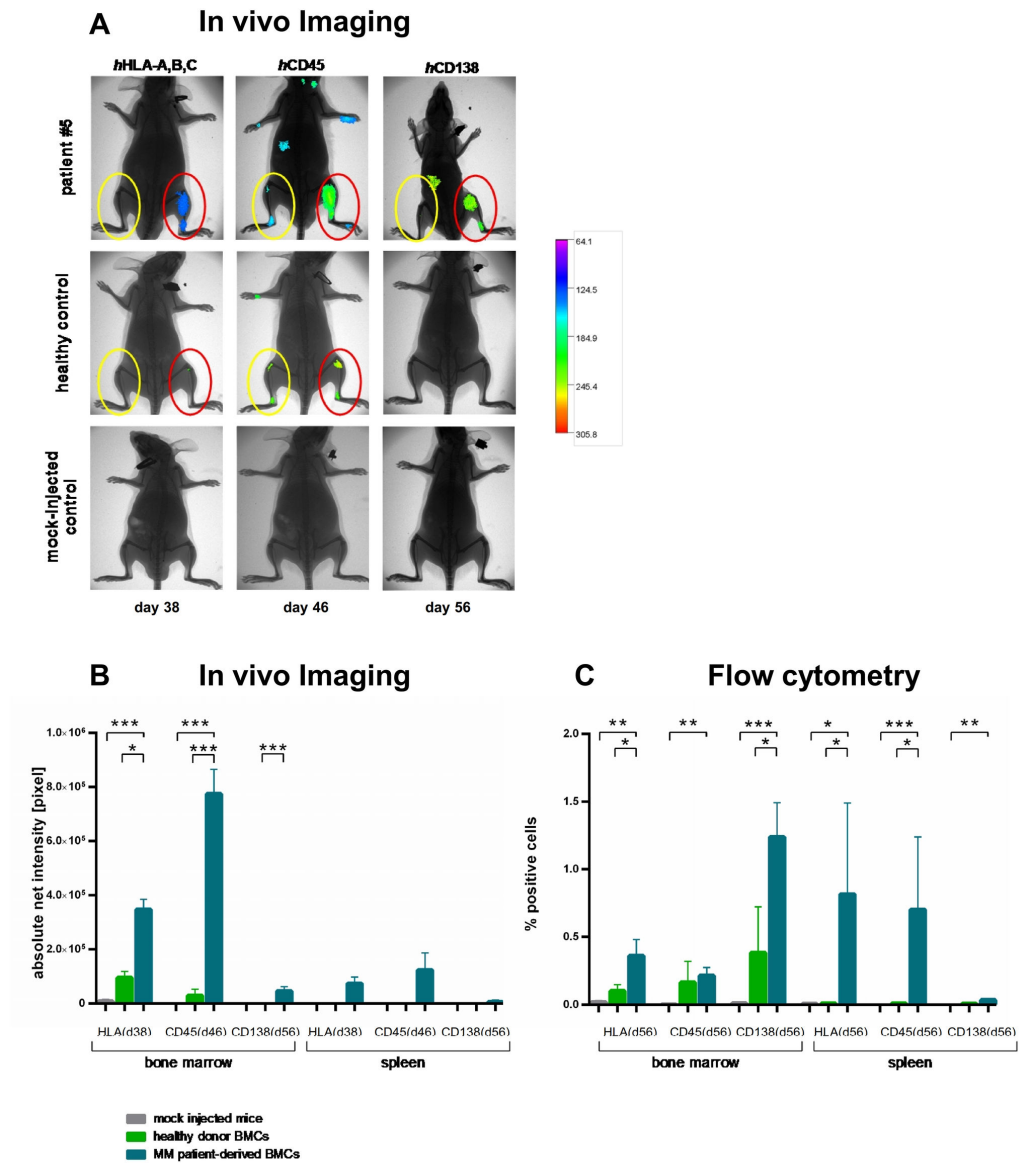
Engraftment of MM patient vs. healthy donor-derived BM cells in NSG and in comparison to mock-injected controls determined by fluorescence-based IVI and flow cytometry. **A. Individual IVI images of NSG mice receiving MM patient or healthy donor-derived BM cells in comparison to mock-injected controls.** As it-injection into NSG induced the most efficacious human cell engraftment, this approach was used for the implantation of MM patient-derived BM cells. IVI was performed using *h*HLA-A,B,C (day 38), *h*CD45 (day 46) and *h*CD138 Abs (day 56) labelled with Alexa750 dye. In addition, animals were x-rayed and the two pictures merged for optimal localization of the fluorescent region. Differences in BM engraftment capacities between mock-injected mice, healthy donor BM or MM-patient-derived BM cells were substantial: use of MM patient-derived BM cells induced sizeable engraftment, both at direct it-injection and to remote sites (upper panel in A). With use of healthy donor BM cells, *h*CD45-and *h*HLA-A,B,C-, but not *h*CD138-Ab-IVI, also revealed minimal engraftment. Therefore, *h*CD138-Ab-IVI revealed most substantial differences between MM, healthy donor and mock-injected cells, showing engraftment with use of MM patient BM cells, but no engraftment with healthy donor or mock-injected cells. **B. Mean net intensity values determined by fluorescence-based IVI of NSG mice receiving MM patient or healthy donor-derived BM cells in comparison to mock-injected controls.** IVI was performed using *h*HLA-A,B,C (day 38), *h*CD45 (day 46) and *h*CD138 Abs (day 56) labelled with Alexa 750 dye. Differences in BM engraftment capacities between mock-injected mice (light grey bars), healthy donor BM (green bars) or MM patient-derived BM-cells (blue bars) were significant (p<0.001). In mock-injected mice, no fluorescence was detected. Healthy donor BM cells could be detected exclusively in the BM, showing no extramedullary engraftment over the entire observation period: After MM patient-derived BM injection, IVI using *h*HLA-A,B,C- and *h*CD45-Ab indicated substantial human engraftment as compared to the *h*CD138-Ab. Flow cytometry helped to confirm the results as shown for a representative patient in A, revealing that CD138 expression on d56 was substantial and significantly higher as compared to mock-injected and healthy donor mice (C). **C. Mean infiltration rates determined by flow cytometry of NSG mice receiving MM patient or healthy donor-derived BM cells in comparison to mock-injected controls.** IVI data from each individual animal was verified by flow cytometry: 56 days after MM patient-derived BM cell injection, BM, PB and spleen specimens were analysed. Human engraftment was defined as >0.08% *h*HLA-A,B,C positivity (= twice the maximum percentage of false-positive cells in mock-injected mice [0.04%]). Injection of BM cells from MM patients resulted in substantially more *h*HLA-A,B,C, *h*CD45 and *h*CD138 positive cells than that of healthy donor BM which was significant for *h*CD138+ cells in the BM, and all three tested markers in the spleen. Furthermore, all investigated markers were significantly higher expressed in BM and spleen of animals bearing MM patient-derived cells compared to mock-injected animals (Student´s t-test, p<0.05). Mean infiltration rates of *h*CD138+ cells in BM, PB and spleen sites were 1.02%, 0.25% and 0.08%, respectively, which appeared fairly substantial, and which were substantially larger than that after healthy donor BM use. Healthy donor-derived BM cells induced very minor BM engraftment (mean infiltration rate of *h*CD138+ cells: 0.39 ± 0.45).

Use of MM patient-derived BM cells substantially enhanced engraftment (Figure 4 + Table 1), which was amplified at direct i.t.-injection and disseminated to remote sites. With use of healthy human BM cells, *h*CD45-and *h*HLA-A,B,C-, but not *h*CD138-Ab-IVI, also revealed minimal engraftment (Figure 4A+B). Of note, *h*CD138-Ab-IVI showed most substantial differences between MM, healthy donor and mock-injected cell engraftment leading to well detectable IVI signals of MM cells and negative IVI signals with healthy donor BM or mock-injected cells (Figure 4A+B).

**Table 1:** Engraftment capacity of MM-patient-derived BM cells vs. healthy donor and mock controls in NSG.

			**# of mice with positive Fluorescence-based *in vivo* imaging signal**			**Flow cytometry^1^**			
**Patient #**	**MM type**	**# of mice**	**HLA ABC**	**CD45**	**CD138**	**HLA ABC**	**CD45**	**CD138**	**CD38**
	**disease stage**	**with disease symptoms**	d38	d46	d56	d56	d56	d56	d56
#1	IgGκ MM IIIA, II SR	4 / 4	4 / 4	4 / 4	4 / 4	+	+	+	+
#2	IgGλ MM IIIA, II SR	8 / 11	4 / 4	4 / 4	4 / 4	+	+	+	-
#3	κ-LC MM IIIA, I HR	1 / 1	n.d.	n.d.	n.d.	+	n.d.	-	n.d.
#4	IgGκ MM IIIA, III SR	1 / 1	n.d.	n.d.	n.d.	+	n.d.	-	n.d.
#5^2^	IgMκ PCL IIIA, III HR	6 / 6	4 / 4	4 / 4	4 / 4	+	+	+	-
#6	IgGκ MM IIIA, II SR	5 / 5	5 / 5	5 / 5	1 / 5	-	+	+	-
#7	IgAκ MM II, I HR	5 / 5	5 / 5	5 / 5	0 / 5	+	+	+	-
#8	IgGκ MM III, II SR	3 / 5	n.d.	3 / 3	2 / 3	+	+	+	+
#9	κ -LC MM IIIA, I SR	3 / 5	3 / 3	2 / 2	1 / 2	-	+	+	-
#10	IgGλ MM IIIA, III SR	3 / 5	5 / 5	5 / 5	3 / 5	+	+	+	-
#11	IgGκ MM IIIA, III	10/10	n.d.	n.d.	10/10³	+³	+³	+³	-³
Control 1	Healthy donor	0 / 7	4 / 7	3 / 7	0 / 7	(+)	(+)	-	-
Control 2	Mock-injection	0 / 8	0 / 3	0 / 3	0 / 3	-	-	-	-

**Abbreviations**:

MM = Multiple Myeloma , HLA-A,B,C = human leucocyte antigen A, B and C; CD45 = Protein tyrosine phosphatase, receptor type, C; CD138 = Syndecan-1;CD38 = ADP-ribosyl cyclase;PCL = Plasma cell leukemia; n.d.= not determined

staging: Durie & Salmon and International staging systemSR=standard-risk via FISH analysis defined as no abnormalities or hyperdiploidy; HR=high-risk: +1q21, del(17p13), immunoglobulin heavy chain gene (IGH) translocation incorporating t(4;14 , t(14;16) and t(14;20).

^1^ Engraftment was determined via flow cytometry analysis with human engraftment defined as equal or larger as 0.08% positivity = +

BM- PB- and spleen-samples were obtained on d56, after primary intratibial injection

^2^ Patient 5 had a plasma cell leukemia, where peripheral blood cells were injected intratibially

^3^ IVI was performed weekly until day 38, flow cytometry was performed on day 38.

### Follow-up analyses of NSG engrafted with patient-derived BM cells: IVI-data

IVI was performed using *h*HLA-A,B,C (day 38), *h*CD45 (day 46) and *h*CD138 Abs (day 56; 2-5 mice/time point; total of 24 animals). Differences in BM engraftment capacities between mock-injected mice, healthy donor or MM-patient-derived BM cells were significant (p<0.001, 1way ANOVA; Fig. 4B). MM patient cell engraftment was predominantly detected in the BM (29/29 mice), but also in the spleen (10/29 mice). Patient-derived cells could be detected over a significantly longer median period of 56 days (n=29) compared to 42 days for healthy BM cells (n=7; p<0.001, Log-rank [Mantel-Cox]-test). Use of *h*HLA-A,B,C (day 38) or *h*CD45 labelled Abs (day 46) appeared to allow more intense human engraftment detection after patient-derived cell injection than with the *h*CD138 Ab (day 56). In mice bearing MM vs. healthy donor cells, quantitative analyses of *h*HLA-A,B,C, *h*CD45 and CD138 fluorescent signals revealed a 3.6-, 26- and 46.000fold higher mean net intensity with MM cells, respectively. 

Healthy donor BM cells could be detected exclusively in the murine BM, showing no extramedullary growth over the entire observation period. Moreover, the *h*CD138 Ab in healthy donor BM bearing mice did not reveal any fluorescent signal (Figure 4A+B). In mock-injected mice, no fluorescence above the background signal was detected.

### Verification of IVI data by flow cytometry 56 days after patient-derived MM cell injection

IVI data from each individual animal was verified by flow cytometry (Figure 4C). Fifty-six days after injection, BM, PB and spleen specimens were analyzed (1-4 animals/patient sample, 19 animals in total). Human cell engraftment was defined as >0.08% *h*HLA-A,B,C positivity observed in mock-injected mice (Figure 4C). MM patient-derived BM, PB and spleen engraftment was observed in 16/19, 18/19 and 8/19 mice, respectively. Injection of BM cells from MM patients vs. healthy donor BM cells resulted in substantially higher *h*HLA-A,B,C, *h*CD45 and *h*CD138 positive cells, which was significant for *h*HLA-ABC+ and *h*CD138+ in the murine BM and for *h*HL*A*-A,B,C+ and *h*CD45+ cells in the spleen (p <0.005, one way ANOVA Figure 4C). Mean infiltration rates of *h*CD138+ cells in BM, PB and spleen were 1.02%, 0.25% and 0.08%, respectively, whereas healthy donor-derived BM cells induced only minor BM engraftment (0.39 ± 0.45%; Figure 4C). The number of *h*CD138+ cells detected in the PB positively correlated with those in the BM and spleen (correlation coefficient: 0.72 and 0.64, respectively; both p <0.005).

### Treatment of L363 tumors with bortezomib and dexamethasone

**Figure 5:**
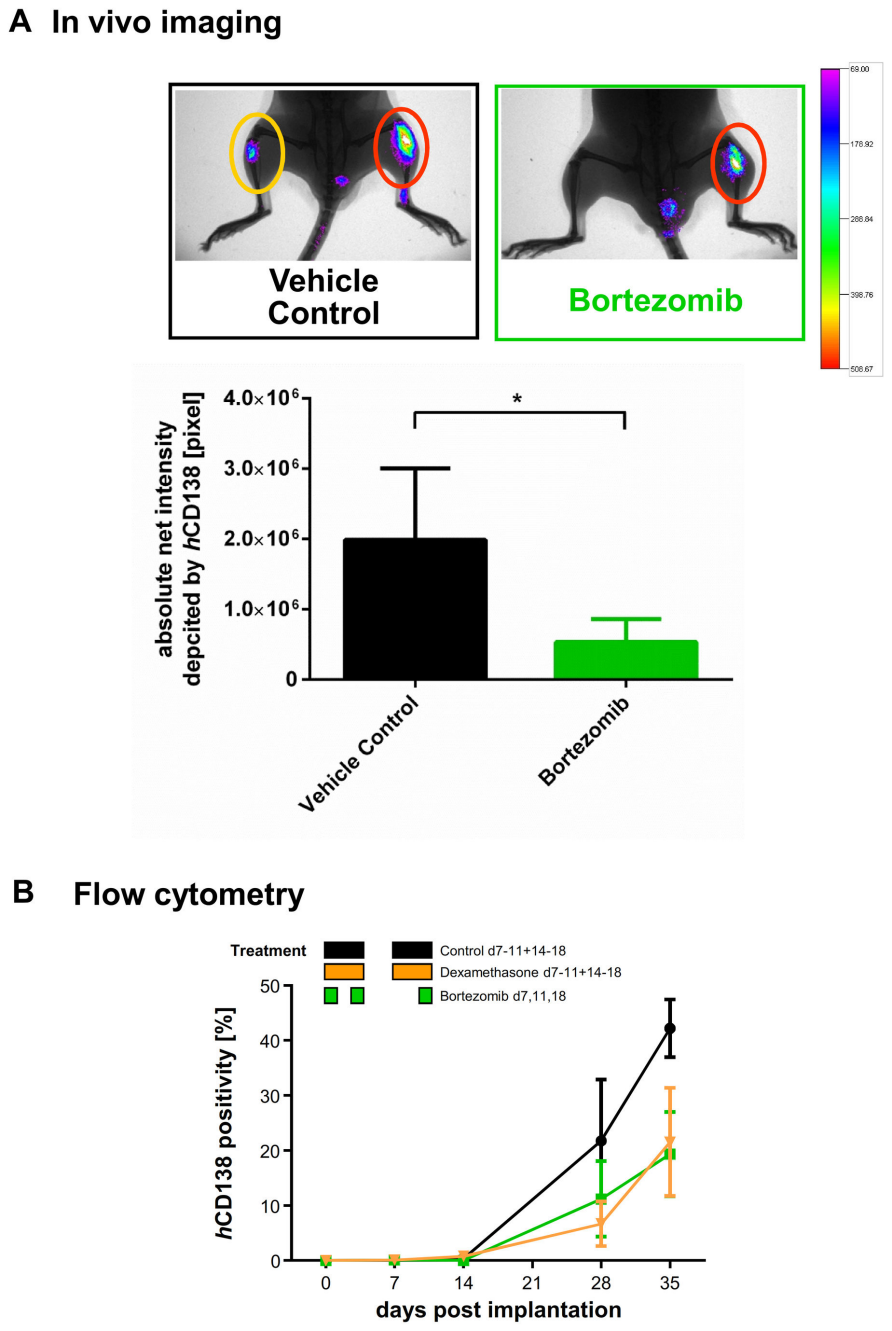
A. Chemosensitivity of L363 cells in NSG against different antimyeloma agents determined by flow cytometry. A total of 24 NSG received it-injections of 2x10^5^ L363 cells: seven days thereafter, 18 mice were randomized into three groups of six animals each. Six additional mice were analysed on the first treatment day (d7) to determine tumor load before the respective therapies. MM cell engraftment was determined by flow cytometry using *h*CD138-Ab (n = 2-3 per group and time point, 4 measurements per group over the time). Black: mice were treated with control vehicle (0.9% NaCl, i.p. d7-11, 14-18). Yellow: mice were treated with 3 mg/kg/d dexamethasone, i.p. (d7-11, 14-18). Green: mice were treated with 0.7 mg/kg/d bortezomib, iv (d7, 11, 18). 7, 14, 28 and 35 days after implantation, tumor cells were assessed via flow cytometry and increased detectably. Analyses on day 35 confirmed that tumor growth was reduced with dexamethasone by 51% and with bortezomib by 66%. **B. Chemosensitivity of L363 cells in NSG against different antimyeloma agents determined by fluorescence-based-IVI.** A total of 15 NSG received it-injections of 2x10^5^ L363 cells: seven days thereafter, mice were randomized into two groups of six and nine animals, respectively. IVI using Alexa750 tagged *h*CD138 antibody was employed to determine tumor engraftment and treatment efficacy. In addition animals were x-rayed and the two pictures merged for optimal localization of the fluorescent region. Tumor growth after bortezomib treatment on day 18 was significantly reduced which was observed both for primary and metastatic sites (Student`s t-test, one-tailed p< 0.05). Error bars depict standard deviation.

Bortezomib and dexamethasone were used as anticancer agents in L363-bearing NSG. Bortezomib was administered with maximum tolerated doses as determined in prior titration studies (Figure S6). L363 cells reliably engrafted the BM (take rate=100%) and distant organs, such as spleen (38%) and rarely liver (8%): 7, 14, 28 and 35 days after implantation, tumor cells were increasingly detectable via IVI (Figure 5A) and flow cytometry (Figure 5B). During the observation period (day 0-35), all mice developed MM symptoms, which were delayed with dexamethasone and bortezomib treatment. 

IVI using Alexa750 tagged *h*CD138 antibody was employed to determine tumor engraftment and treatment efficacy. Tumor growth after bortezomib treatment on day 18 was again markedly reduced which reached statistical significance (Figure 5B, Student`s t-test, one-tailed p <0.05).

Flow cytometry guided detection of hCD138+ cells in the BM (Figure 5B) and spleen (not shown) revealed lesser *h*CD138+ cells in mice treated with dexamethasone and bortezomib. As compared to control animals, dexamethasone- and bortezomib-treated mice showed 51% and 66% less *h*CD138 positive cells within the BM on day 35, respectively, which - although this did not reach significance (Student`s t-test, two-tailed) - confirmed that primary tumor growth was remarkably reduced. Similarly, we observed substantial downregulation by dexamethasone and bortezomib treatment via *h*HLA-ABC assessment in BM and spleen (data not shown).

### Chemosensitivity of patient-derived MM cells in NSG against bortezomib and dexamethasone

**Figure 6:**
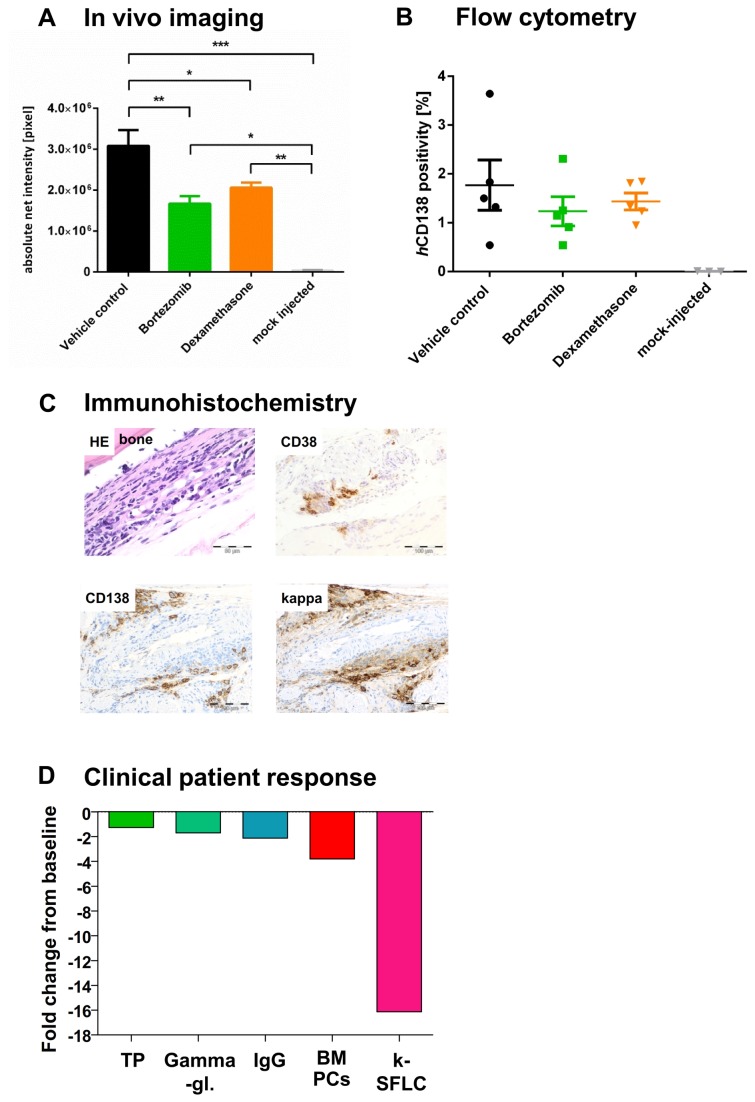
Chemosensitivity of patient-derived MM cells in NSG against different antimyeloma agents determined by fluorescence-based-IVI (A-B) and flow cytometry (C) compared to clinical outcome of the donor patient (D). A total of 30 NSG received it-injections of 2x10^6^ patient derived MM cells: 11 days thereafter, mice were randomized into three groups of ten animals each. Black: mice were treated with control vehicle (0.9% NaCl, i.p. d11-15, 17-22). Yellow: mice were treated with 3 mg/kg/d dexamethasone, i.p. (d11-15, 17-22). Green: mice were treated with 0.7 mg/kg/d bortezomib, iv (d11, 15, 19, 22). **A. Tumor load determination using fluorescence based IVI.** IVI using Alexa750 tagged *h*CD138 antibody was employed to determine tumor engraftment and treatment efficacy from day 11 (first day of treatment) on once weekly. In addition animals were x-rayed and the two pictures merged for optimal localization of the fluorescent region. Non-tumor bearing animals were treated accordingly and used as negative control. Error bars depict standard deviation. Determination of net intensity over time revealed a reliable engraftment of patient-derived MM cells over time as compared to mock-injected mice. Treatment with bortezomib or dexamethasone reduced tumor progression compared to untreated control. Tumor growth after bortezomib and dexamethasone treatment on day 26 was significantly reduced by 40% and 22%, respectively, which was observed both for primary and metastatic sites (one way ANOVA, p<0.0001). **B. MM cell engraftment in the BM determined by flow cytometry.** MM cell engraftment in the BM was determined by flow cytometry using *h*CD138-Ab on the last experiment day (d=38, n=5 per group). Analyses confirmed that tumor growth was reduced with dexamethasone and bortezomib. **C. Immunhistochemical stainings of a femur from a NSG mouse inoculated with MM patient-derived BM cells.** Formalin fixed and paraffin embedded long bones from tumor bearing mice were stained with HE, anti-human CD38-, CD138- and kappa-Ab. The patient's kappa/CD38/CD138 triple-positive BM cells were well detectable in NSG mice 35 days after tumor cell inoculation. IHC analysis for hCD138 expression confirmed IVI and flow cytometry results as depicted in 6A+B. **D. Specific response parameters in the patient after treatment.** Specific response parameters in the patient after treatment. improved after three cycles of bortezomib, cyclophosphamide and dexamethasone (VCD) induction. A decline in his total protein (TP), gamma-globuline fraction (g-Gl), IgG, BM plasma cells (BM-PCs) and kappa-light chains (serum free light chains, [k-SFLC]) was induced as determinants of VCD response. Results are given as fold decreases from baseline levels.

Chemosensitivity of patient-derived MM cells against bortezomib and dexamethasone was depicted in i.t.-injected NSG mice. Determination of net intensity using Alexa750 tagged *h*CD138 antibody over time revealed a reliable engraftment of patient derived MM cells as compared to mock-injected mice. Treatment with bortezomib or dexamethasone reduced tumor progression compared to untreated control (Figure 6A). Evaluation of tumor load via IVI determined a significantly reduced tumor load due to bortezomib (40%) and dexamethasone (22%) injection in primary as well as metastatic sites (Figure 6A, one way ANOVA, p<0.0001) on day 26. Beyond that, MM cell engraftment in the BM was determined by flow cytometry using *h*CD138-Ab on the last experiment day (d38, n=5 per group), depicting reduced tumor growth with bortezomib and dexamethasone (Figure 6B). Results from IVI and flow cytometry were confirmed by immunohistochemical staining of femura from tumor bearing NSG mice: the patient's kappa/CD38/CD138 triple-positive BM cells were well detectable in NSG mice 38 days after tumor cell inoculation (Figure 6C). Treatment response in our NSG model was in line with the patient's excellent response after three cycles of VCD (bortezomib, cyclophosphamide, dexamethasone) induction (Figure 6D), highlighting the utility of developing this model into a monitoring system to personalize therapeutic interventions. 

## Discussion

Various myeloma mouse models have been reported, including human MM xenografts [6]. Most have tested transplantation and engraftment of established murine or human cell lines in syngeneic or immunocompromised mice, respectively. While subcutaneous or i.p. cell-line based xenograft models are useful for relatively inexpensive screening of novel drugs, they are not considered ideal, because they do not replicate the human BM milieu. Models that recapitulate the *in vivo* growth of primary MM cells in a human (SCID-hu) or humanized (SCID-synth-hu) murine recipient, have been valuable for the investigation of new compounds, but rely on the availability of fetal bone chips and synthetic bone scaffolds [12]. Similarly to the SCID-hu model, rabbit bone implanted into SCID mice has allowed successful engraftment of unsorted BM cells from MM patients [13]. If SCID mice are implanted with human fetal bone chips (SCID-hu mice), INA-6 cells engraft successfully therein. This s.c INA-6 inoculation into SCID-hu mice has allowed engraftment of MM cells, whereas this is futile in SCID mice that have not been given implants of human fetal bone. Although the SCID-hu model remains relevant, it has some limitations: these include the availability of fetal bone chips, the heterogeneity of implanted human bone and differences between the environment of the fetal and the elderly patients' bone. The SCID-synth-hu model overcomes these limitations, as it allows growth of primary MM cells within a 3D biosynthetic implant coated with adult BM stroma. While it is a unique model for translational research and recapitulates the growth of MM cells within the specific human BM microenvironment, the SCID-synth-hu model also bears challenges, as it relies on a polymeric scaffold which is eagerly investigated for its reproducibility of BM stroma and primary MM cell growth. Groen et al. recently demonstrated the need for a human niche to achieve efficient engraftment of patients' myeloma cells: they described a human-mouse hybrid model that allows engraftment and outgrowth of normal and malignant hematopoietic progenitors by implementing a technology for generating a human BM environment. Using luciferase gene marked patient-derived MM cells and bioluminescent imaging, MM outgrowth and treatment effects could be monitored. Advantages of this hybrid model are that it allows to investigate the microenvironmental influence on benign and malignant hematopoietic development and to use this for personalized therapeutic strategies. Albeit this model bears novel opportunities, the comparison of NSG and NOD/SCID models has not been systematically conducted for various preclinical scenarios. While i.t.-injection of RPMI8226 cells into nude mice has been demonstrated to induce focal osteolysis, this strategy has remained largely untested with different permanent cell lines or primary MM cells in other mouse strains.

In the present study, growth kinetics of two MM cell lines and patient-derived MM cells were systematically investigated in different mouse strains (NOD/SCID, αCD122 pre-treated NOD/SCID and NSG). Our data support the preclinical rationale to use i.t.-injected NSG, since they closely resemble clinical MM with respect to symptoms, disseminated disease sites and response to anticancer treatment. An essential requirement for human MM cell growth within the murine environment was the depletion of NK cells: take, survival and infiltration rates at the injection site and in distant organs were increased with lack of the NK cell activity, using either NSG or αCD122 pre-treated NOD/SCID. These observations are in line with previous data reporting that murine NK cells prohibit the engraftment in murine sites and induce antitumor activity [14,15]. Comparison of NSG and αCD122-Ab-treated NOD/SCID revealed enhanced growth kinetics in NSG. Notably disseminated tumor sites were exclusively observed in i.t.-injected NSG. This may be explained by the profound impairment of multiple immunological functions observed in NSG recipients. Furthermore, IL-2Rγchain (CD132), in contrast to the IL2Rβ chain (CD122) does not only interact with IL-2, but is a common component of several interleukin receptors, playing a crucial role in immunological response mechanisms [16]. Of note, the engraftment within the 'i.t.-NOD/SCID + CD122' model was lower compared to the 'b.i. NOD/SCID+CD122' and also compared to 'i.t. NSG' approach, possibly related to the NK block induced by CD122 Ab treatment that is more efficient in the subcutaneously implanted long bone than in the murine BM due to the enhanced vascularisation of the former. That our i.t. approach in NSG was more efficacious than the i.v.-injection may relate to the fact that MM cells circulate in the blood prior to their BM homing. Thus, any host resistance preventing the cells from reaching their destination within the BM microenvironment hampers human myeloma growth. Our data are consistent with the observations of McKenzie who described enhanced engraftment of human myeloid and lymphoid cells, when cells were i.t.-injected [17].

In NOD/SCID with functional NK cells, our use of subcutaneously injected juvenile long bone implants served as a tumor cell niche and was superior to the i.t.- or i.v.-injection. This seems related to the enhanced neovascularisation activity induced by the implantation of bone tissue. Nevertheless, due to the more disseminated growth of the MM cells in i.t.-injected NSG, the latter was the clinically more relevant model. Determination of human MM cell engraftment was readily detectable via *h*HLA-ABC or *h*CD138 expression by flow cytometry or fluorescence-based IVI and, at best, if L363 had been i.t.-injected into NSG. Due to the lack of paraprotein production within the L363 model and the need of a highly sensitive ELISA technique for xenograft assays in general [18], our study did not include serum paraprotein determination as a measurement for tumor load rather than focusing on IVI, flow cytometry and immunohistochemistry analyses which allowed us to follow the dynamics of MM growth. IVI in comparison to serum analyses enabled us to distinguish the engraftment at primary sites and spreading to secondary sites. The suitability of i.t.-implanted NSG was further investigated using MM patient-derived BM cells. Compared to healthy BM- and mock-injected controls, MM cell engraftment was substantially enhanced, predominantly in the murine BM. Moreover, i.t.-implanted mice developed typical disease symptoms. In line with others [19], who demonstrated that unseparated BM cells grow equally well to purified plasma cells and that accessory cells support sustained myeloma growth in suitable murine models, we did not separate BM cells from MM patients. Apart from that, their median BM infiltration rate was high and cell separation would have led to substantial cell loss. Fifty-six days post-injection, patient-derived tumor cells could be detected via flow cytometry analysis in all injected animals. Our data confirmed prior observations of patient-derived-MM cells in SCID-rab- [13] or SCID-hu-models [20], where directly injected bone implants or i.v.-injection induce MM symptoms, only after much longer observation times. We postulate that the combination of our i.t.-approach and use of NSG led to a direct contact of patient MM cells with the murine BM milieu, resulting in enhanced engraftment of these cancer cells. This was confirmed in our i.t.-NSG model in multiple treatment experiments using L363 or patient-derived MM cells with dexamethasone and bortezomib that exhibited a disease pattern and treatment response closely mimicking the clinical situation, highlighting the utility of developing this model as a monitoring system to personalize therapeutic interventions. 

Our fluorescence-based-IVI using a fluorophore in the near-infra-red range proved to be a time and animal saving analysis that allowed monitoring of MM growth *in vivo*. Similar observations have been reported by other groups examining different models of solid tumors and leukemia [21]. That a convenient, reliable and sensitive tracking of myeloma cells is indeed valuable with whole body imaging techniques in living animals has been demonstrated by various groups [22,23]. Bioluminescence and fluorescence imaging are widely used for determination of tumor load in preclinical *in vivo* studies. The outstanding advantage of real-time whole-body-imaging is the opportunity to monitor individual experimental mice over prolonged time periods, which is not possible with established techniques such as flow cytometry or immunohistochemistry [24]. 

Despite a plethora of luciferase reporter genes in use, monitoring multiple events simultaneously in small animals is also challenging, partly attributable to the lack of optimization of cell reporter expression as well as too much spectral overlap of colour-coupled reporter genes [25]. Additionally, the introduction of a foreign reporter protein may alter tumor development [26].

Our model can be criticized, because MM cells from patients readily engrafted and disseminated, albeit negligible healthy BM cells were also detected therein. Moreover, our fluorescence-IVI-Abs, *h*HLA-A,B,C, *h*CD45 and *h*CD138, had to be assessed at different time points, making their comparison challenging and suggesting that *h*CD138 is preferentially used earlier, as performed here in the treatment experiment using patient derived BM cells. Although marker downregulation induced by tumor microenvironment has previously been described for *h*CD138 by different groups [27,28] and proved to be in line with our own data (as shown in Figure S5) *h*CD138 turned out to be a reliable and sensitive marker for tumor cell localization in vivo. Flow cytometry results verified dissemination of *h*CD138+ MM cells in BM, PB and spleen sites, which were significantly higher in the BM than after inoculation of healthy donor cells, and undetectable with use of healthy donor BM cells in PB and spleen sites. Moreover, our model uses the murine BM microenvironment instead of a reconstructed human BM milieu as recently published [11]. This has to be taken into account when it comes to the evaluation of drugs that specifically target the human microenvironment, where human-mouse hybrid models provide exceptional opportunities. Future perfection of our i.t. NSG approach may include exploitation of conditioned mice, utilization of CD138-selected MM cells and use of human mesenchymal stem cells to create a humanized environment. A deeper insight into the biology of our model will also be provided by analyzing the malignant clone by FISH and by follow-up studies in secondary recipients. In summary, our data demonstrate that i.t.-injected NSG mimic clinical MM disease with respect to symptoms, engraftment of clonogenic plasma cells into the BM and tumor dissemination. The confirmation of MM cell line engraftment with primary patient-derived BM cells further validated that our model constitutes a valuable *in vivo* tool for monitoring responses to anticancer agents.

## Supporting Information

Figure S1 **Cell binding studies with Alexa750 labeled *h*HLA-A,B,C, *h*CD138 and *h*CD45 antibodies to human L363 and RPMI8226 cells.** Cell binding studies with Alexa750-labeled antibodies (AF750-Abs) at a single concentration of 5µg/ml were carried out in triplicates using L363 and RPMI8226 cells (1×10^6^ cells per experiment). Cells were incubated in the presence of the AF750-Abs for 2hrs at room temperature (=w/o blocking). For nonspecific uptake control experiments, the cells were first saturated by incubating with excess of non-fluorescent Abs (100µg/ml) for 0.5h (=with blocking). After washing with PBS, the fluorescent intensity was measured with a Kodak Image Station *in* *vivo* FX (**A**). The Mann-Whitney U *t* test was used to determine statistical differences in the cell binding of the AF750-Abs to both L363 and RPMI8226 cells (p<0.05). Murine bone marrow (BM) and spleen cells were used as controls and treated as described. The cell binding of the AF750-Abs was substantial with *h*HLA-A,B,C and *h*CD138 and low with *h*CD45. This was in line with the FACS expression profile of the investigated cell lines (**B**). When cells were incubated with an excess of unlabeled antibody to saturate the receptors, the percentage of total AF750-mAb bound to the tested MM cells substantially decreased for *h*HLA-A,B,C and *h*CD138, both with use of L363 and RPMI cells (p< 0.001, one way ANOVA). Neither murine BM nor murine spleen cells (data not shown) revealed binding to the investigated antibodies, therefore the chosen antibodies exposed excellent specificity for human, but not murine cells.
(TIF)

Figure S2 **Comparison of fluorescence signal *invivo* before and after antibody treatment and in response to different previous labellings.** Injection of the three different antibodies coupled to the same fluorochrome was performed in the same chronological order and time course as in the main analysis (e.g. results of Figure 4). L363-bearing NSG mice were used to induce similar human tumor cell engraftment in the examined animals. Differences in net intensity - before and after injection of the respective antibody *h*HLA, (**A**) *h*CD45 (**B**) and *h*CD138 (**C**) to detect human tumor cell engraftment in NSG mice on day 14 were statistically significant for all three antibodies in the BM and spleen of tumor bearing mice (unpaired t-test). Moreover, the net intensity of the IVI signal was equally high in animals receiving no pretreatment with labeled antibody (**A**), *h*HLA-A,B,C (**B**) or prior *h*HLA-A,B,C and *h*CD45 injection (**C**).
(TIF)

Figure S3 **Comparison of the residual fluorescence signal intensity (background) *invivo* before antibody treatment and after different previous labelings.** Injection of the three different antibodies coupled to the same fluorochrome was performed in the same chronological order and time course as in the main analysis (see e.g. Figure 4). L363-bearing NSG mice were again used as described. The determined residual net intensity (=background) in animals pre-treated with different antibodies was equal to animals which had never received a fluorochrome coupled antibody, both in the BM and spleen (one way ANOVA).
(TIF)

Figure S4 **A. Determination of the detection limit of the IVI assay *invitro* using various cell numbers.** 1x10^6^ L363 cells were used as positive control and a CD138-negative cell line RKO (1x10^6^ cells each) was used as a negative control, revealing a background signal net intensity of < 1x10^5^ pixel/region of interest. Cells were incubated in the presence of the *h*CD138- AF750-antibody for 2hrs at room temperature (=w/o blocking). For nonspecific uptake control experiments, the cells were first saturated by incubating with excess of non-fluorescent Abs (100µg/ml) for 0.5h (=with blocking). After washing with PBS, the fluorescent intensity was measured with a Kodak Image Station *in* *vivo* FX. The cell binding assay clearly indicated a specific signal when at least 5x10^5^ CD138 expressing cells (=L363) were used. **B. Determination of the detection limit of the IVI assay *invivo* using various cell numbers.** L363 cells were injected either into the BM (tibia) or into the spleen. Fluorescence-based whole body and organ imaging were performed immediately after tumor cell injection. After antibody injection, mice were anesthetized by isoflurane inhalation and images were taken with a Kodak *in* *vivo* imaging system (=whole body imaging). Immediately following the whole body imaging, animals were sacrificed and injected tibia or spleen were removed and an additional image was taken (= organ imaging). The determined detection limit was 5x10^5^ cells for spleen and BM (tibia), albeit the signal in the spleen was weaker compared to the BM. This is clearly related to the fact that the spleen signal has to penetrate a significant amount of tissue, whereas the signal source from femur and tibia in a dorsal-ventral imaging setting is close to the surface.
(TIF)

Figure S5 **A. Surface marker expression of L363 and RPMI8226 cells prior to (=d0) and after injection into immuncompromised NSG mice (=d35).** Surface marker expression of *h*HLA-A,B,C, *h*CD138 and *h*CD45 was determined by flow cytometry in 4 different experiments before (d0) and after propagation in immuncompromised mice: for the latter, i.t.-injected NSG mice were used and HLA-ABC, CD138 and CD45 assessed on day 35 after injection (=d35). In both L363 and RPMI8226, *h*HLA-A,B,C and *h*CD138 were substantially expressed on d0, whereas CD45 was significantly lower. When assessed in i.t.-injected NSG mice, both *h*HLA-ABC and *h*CD138 were substantially lower, but still well detectable. The low expression of *h*CD45 *in* *vitro* (2% for L363 and 10% for RPMI8226) was markedly downregulated, when cells were propagated *in* *vivo*. **B. Comparison of surface marker expression profiles (HLA-ABC, CD138 and CD45) of human L363 and RPMI8226 cells to myeloma patient- and healthy donor-derived cells (=d0).** Surface marker expression of MM patients' vs. healthy donor BM cells prior to injection into immuncompromised mice: *h*HLA-A,B,C, *h*CD138 and *h*CD45 expression was determined by flow cytometry in 10 MM patient specimens (9 BM and 1 PB form a patient with plasma cell leukemia) as compared to healthy donor BM cells. This revealed most substantial differences for CD138 which was significantly enhanced in MM patients' specimen and low in healthy donor BM cells. The surface marker expression profile of MM patient specimens showed resemblance to both RPMI and L363 cells.
(TIF)

Figure S6 **Body weight changes of non-tumor bearing NSG mice under different concentrations of bortezomib treatment.** Black: vehicle control (0.9% NaCl, iv (d0,4,7,11); red: 0.7mg/kg/d Bortezomib, i.v. (days 0,4,11); blue: 1.3mg/kg/d Bortezomib, i.v. (days 0,7). Bortezomib treatment at the higher dose level of 1.3mg/kg could be given on day 0 + 8, but had to be omitted on day 4 and 11 due to median body weight losses of 19% and 20%, respectively. The lower dose of 0.7mg/kg bortezomib could be applied more frequently on day 0, 4 and 11, because this resulted in a median body weight loss of only 6%. Since mice had not fully recovered on day 8, the day 8-treatment was omitted to ensure survival of all animals, resulting in three single, safely administered bortezomib injections on day 0, 4, 11 as depicted in blue. At the end of the observation period (23 days after the first injection), bortezomib-treated, non-tumor bearing mice had reached their initial body weight, almost comparable to untreated controls that achieved a 1.2-fold increase over their initial body weight.
(TIF)

Table S1 **Survival of immunocompromised mice implanted with L363 or RPMI8226 MMCLs.**
(DOC)

Table S2 **A**. **Individual MM patient and sample characteristics; B. Summarized clinical MM patient characteristics.**
(DOC)
