# Supplementary figures and images for: Intratibial Injection of Human Multiple Myeloma Cells in NOD/SCID IL-2Rγ(Null) Mice Mimics Human Myeloma and Serves as a Valuable Tool for the Development of Anticancer Strategies
Source: PLoS One. 2013 Nov 6;8(11):e79939. doi: 10.1371/journal.pone.0079939 (PMC3819303; doi:10.1371/journal.pone.0079939)

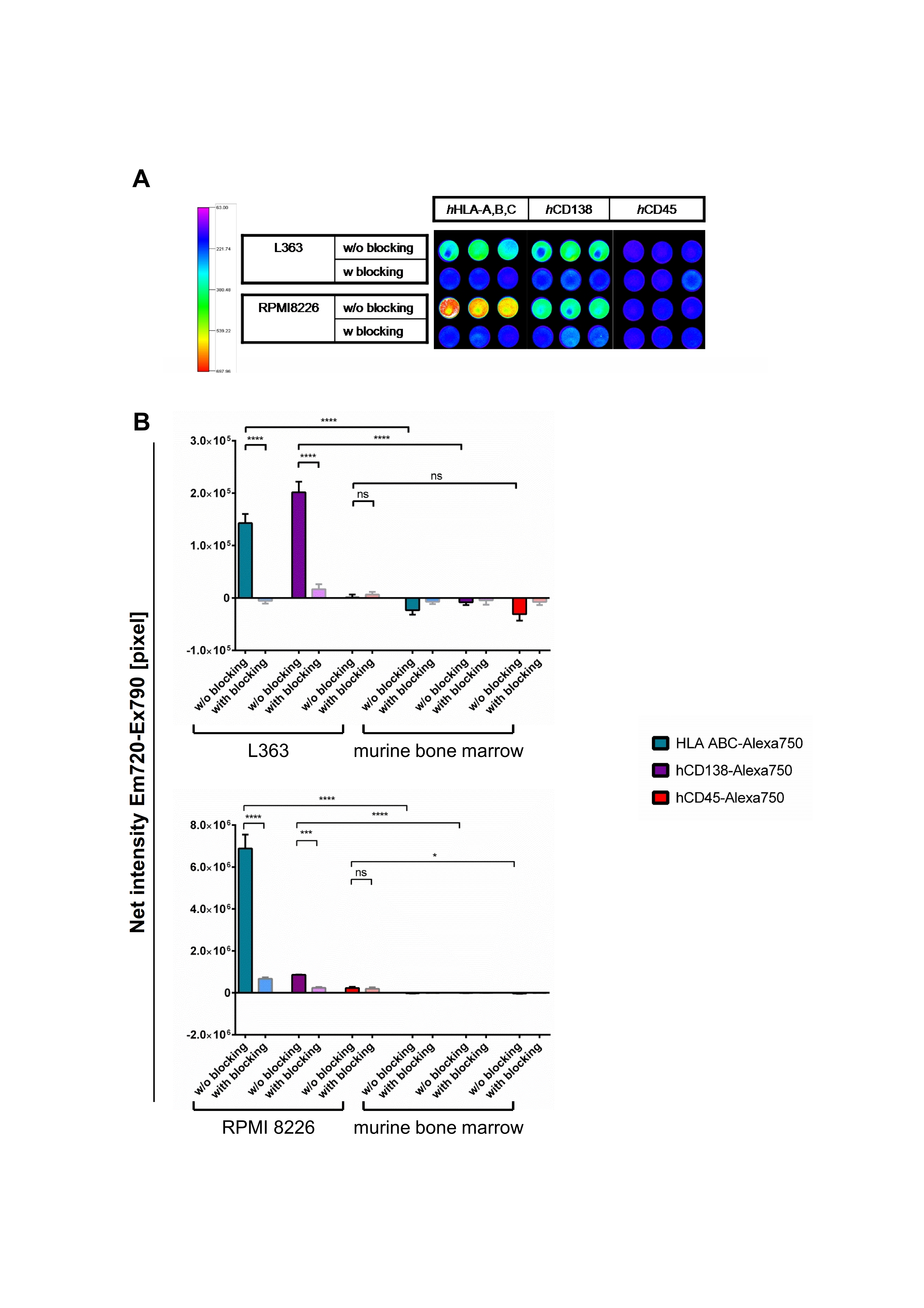

Supplement: Figure S1 — Cell binding studies with Alexa750 labeled hHLA-A,B,C, hCD138 and hCD45 antibodies to human L363 and RPMI8226 cells. Cell binding studies with Alexa750-labeled antibodies (AF750-Abs) at a single concentration of 5µg/ml were carried out in triplicates using L363 and RPMI8226 cells (1×10^6^ cells per experiment). Cells were incubated in the presence of the AF750-Abs for 2hrs at room temperature (=w/o blocking). For nonspecific uptake control experiments, the cells were first saturated by incubating with excess of non-fluorescent Abs (100µg/ml) for 0.5h (=with blocking). After washing with PBS, the fluorescent intensity was measured with a Kodak Image Station in vivo FX (A). The Mann-Whitney U t test was used to determine statistical differences in the cell binding of the AF750-Abs to both L363 and RPMI8226 cells (p<0.05). Murine bone marrow (BM) and spleen cells were used as controls and treated as described. The cell binding of the AF750-Abs was substantial with hHLA-A,B,C and hCD138 and low with hCD45. This was in line with the FACS expression profile of the investigated cell lines (B). When cells were incubated with an excess of unlabeled antibody to saturate the receptors, the percentage of total AF750-mAb bound to the tested MM cells substantially decreased for hHLA-A,B,C and hCD138, both with use of L363 and RPMI cells (p< 0.001, one way ANOVA). Neither murine BM nor murine spleen cells (data not shown) revealed binding to the investigated antibodies, therefore the chosen antibodies exposed excellent specificity for human, but not murine cells. (TIF) [file pone.0079939.s001.tif]

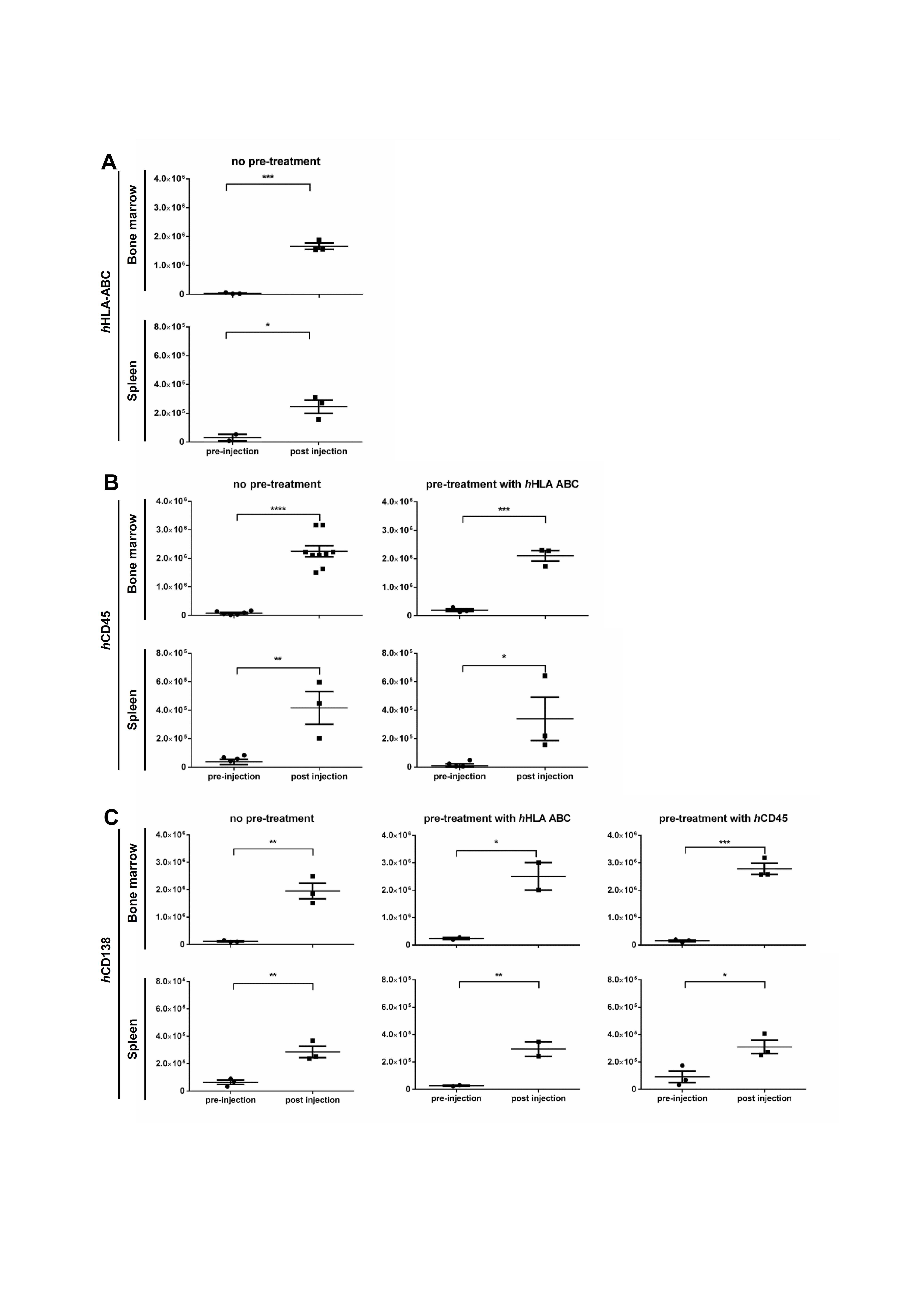

Supplement: Figure S2 — Comparison of fluorescence signal invivo before and after antibody treatment and in response to different previous labellings. Injection of the three different antibodies coupled to the same fluorochrome was performed in the same chronological order and time course as in the main analysis (e.g. results of Figure 4). L363-bearing NSG mice were used to induce similar human tumor cell engraftment in the examined animals. Differences in net intensity - before and after injection of the respective antibody hHLA, (A) hCD45 (B) and hCD138 (C) to detect human tumor cell engraftment in NSG mice on day 14 were statistically significant for all three antibodies in the BM and spleen of tumor bearing mice (unpaired t-test). Moreover, the net intensity of the IVI signal was equally high in animals receiving no pretreatment with labeled antibody (A), hHLA-A,B,C (B) or prior hHLA-A,B,C and hCD45 injection (C). (TIF) [file pone.0079939.s002.tif]

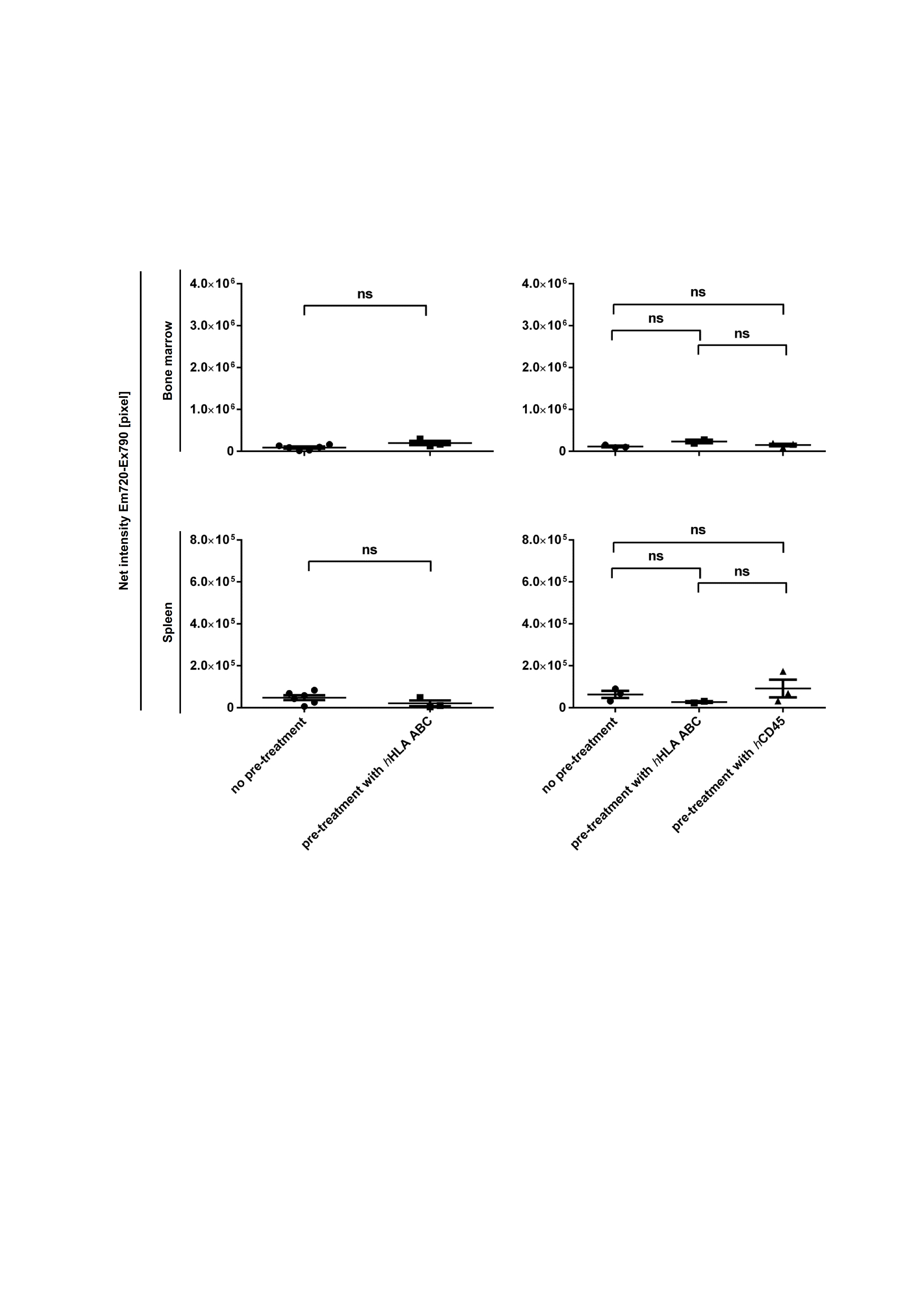

Supplement: Figure S3 — Comparison of the residual fluorescence signal intensity (background) invivo before antibody treatment and after different previous labelings. Injection of the three different antibodies coupled to the same fluorochrome was performed in the same chronological order and time course as in the main analysis (see e.g. Figure 4). L363-bearing NSG mice were again used as described. The determined residual net intensity (=background) in animals pre-treated with different antibodies was equal to animals which had never received a fluorochrome coupled antibody, both in the BM and spleen (one way ANOVA). (TIF) [file pone.0079939.s003.tif]

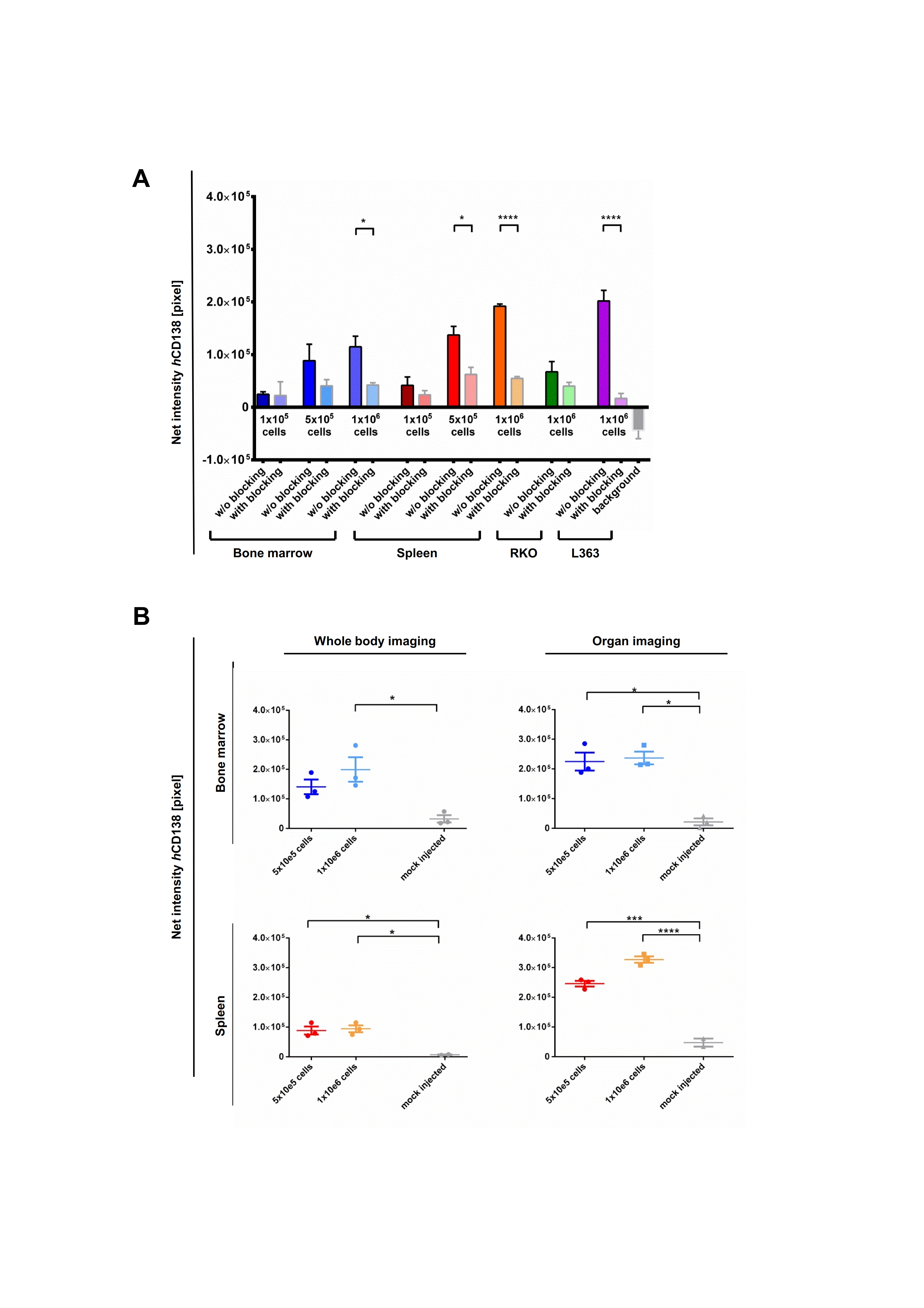

Supplement: Figure S4 — A. Determination of the detection limit of the IVI assay invitro using various cell numbers. 1x10^6^ L363 cells were used as positive control and a CD138-negative cell line RKO (1x10^6^ cells each) was used as a negative control, revealing a background signal net intensity of < 1x105 pixel/region of interest. Cells were incubated in the presence of the hCD138- AF750-antibody for 2hrs at room temperature (=w/o blocking). For nonspecific uptake control experiments, the cells were first saturated by incubating with excess of non-fluorescent Abs (100µg/ml) for 0.5h (=with blocking). After washing with PBS, the fluorescent intensity was measured with a Kodak Image Station in vivo FX. The cell binding assay clearly indicated a specific signal when at least 5x10^5^ CD138 expressing cells (=L363) were used. B. Determination of the detection limit of the IVI assay invivo using various cell numbers. L363 cells were injected either into the BM (tibia) or into the spleen. Fluorescence-based whole body and organ imaging were performed immediately after tumor cell injection. After antibody injection, mice were anesthetized by isoflurane inhalation and images were taken with a Kodak in vivo imaging system (=whole body imaging). Immediately following the whole body imaging, animals were sacrificed and injected tibia or spleen were removed and an additional image was taken (= organ imaging). The determined detection limit was 5x10^5^ cells for spleen and BM (tibia), albeit the signal in the spleen was weaker compared to the BM. This is clearly related to the fact that the spleen signal has to penetrate a significant amount of tissue, whereas the signal source from femur and tibia in a dorsal-ventral imaging setting is close to the surface. (TIF) [file pone.0079939.s004.tif]

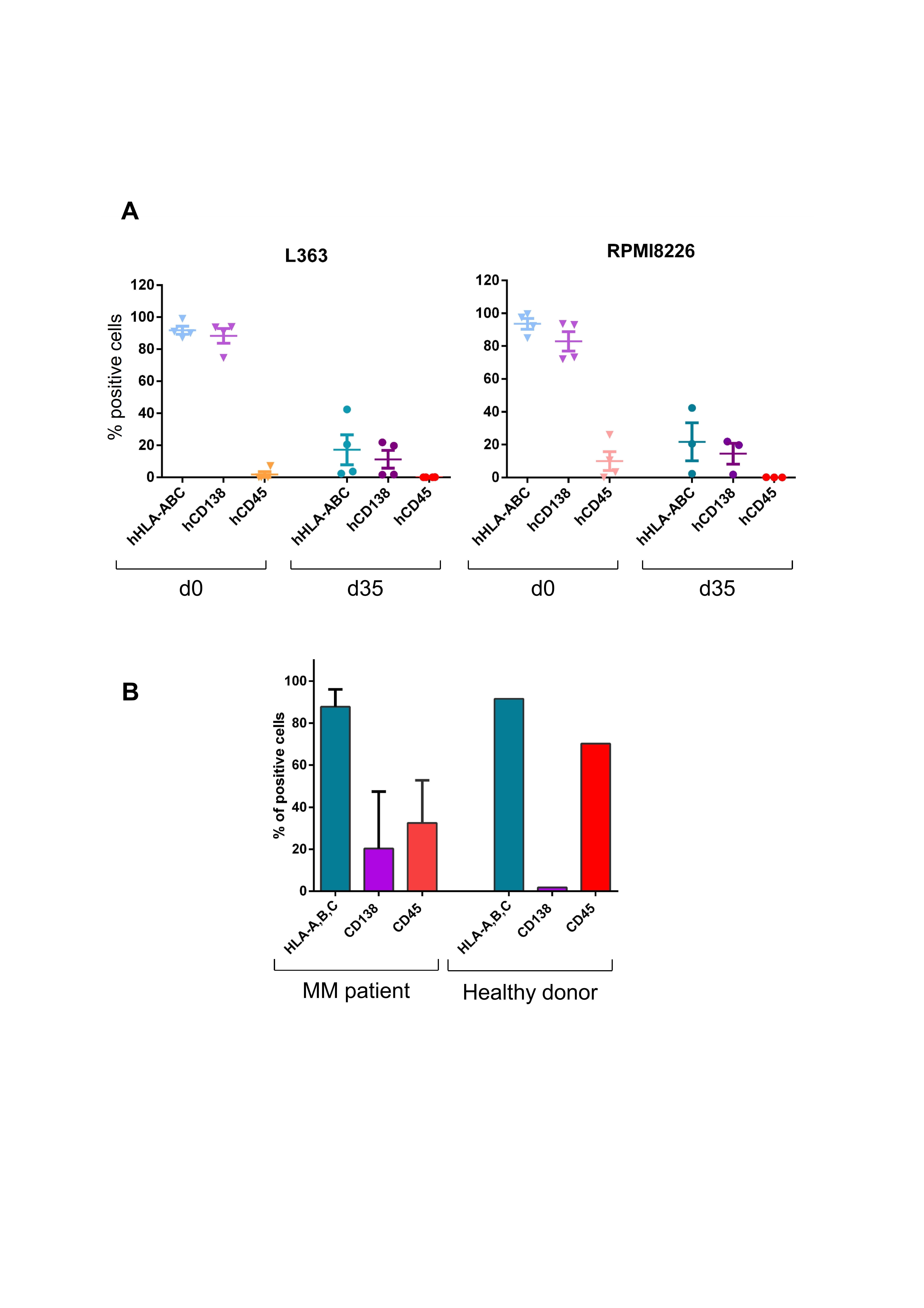

Supplement: Figure S5 — A. Surface marker expression of L363 and RPMI8226 cells prior to (=d0) and after injection into immuncompromised NSG mice (=d35). Surface marker expression of hHLA-A,B,C, hCD138 and hCD45 was determined by flow cytometry in 4 different experiments before (d0) and after propagation in immuncompromised mice: for the latter, i.t.-injected NSG mice were used and HLA-ABC, CD138 and CD45 assessed on day 35 after injection (=d35). In both L363 and RPMI8226, hHLA-A,B,C and hCD138 were substantially expressed on d0, whereas CD45 was significantly lower. When assessed in i.t.-injected NSG mice, both hHLA-ABC and hCD138 were substantially lower, but still well detectable. The low expression of hCD45 in vitro (2% for L363 and 10% for RPMI8226) was markedly downregulated, when cells were propagated in vivo. B. Comparison of surface marker expression profiles (HLA-ABC, CD138 and CD45) of human L363 and RPMI8226 cells to myeloma patient- and healthy donor-derived cells (=d0). Surface marker expression of MM patients' vs. healthy donor BM cells prior to injection into immuncompromised mice: hHLA-A,B,C, hCD138 and hCD45 expression was determined by flow cytometry in 10 MM patient specimens (9 BM and 1 PB form a patient with plasma cell leukemia) as compared to healthy donor BM cells. This revealed most substantial differences for CD138 which was significantly enhanced in MM patients' specimen and low in healthy donor BM cells. The surface marker expression profile of MM patient specimens showed resemblance to both RPMI and L363 cells. (TIF) [file pone.0079939.s005.tif]

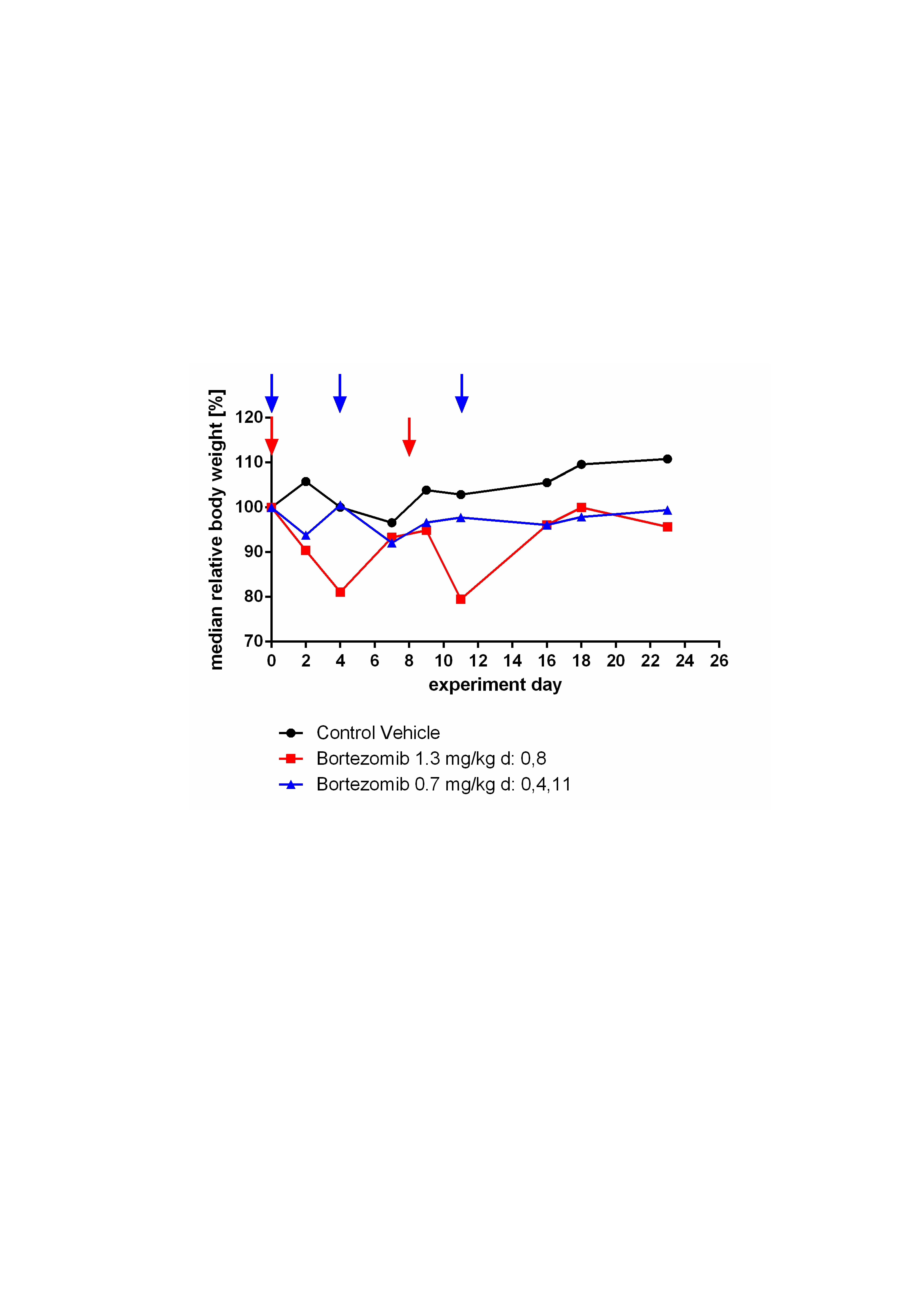

Supplement: Figure S6 — Body weight changes of non-tumor bearing NSG mice under different concentrations of bortezomib treatment. Black: vehicle control (0.9% NaCl, iv (d0,4,7,11); red: 0.7mg/kg/d Bortezomib, i.v. (days 0,4,11); blue: 1.3mg/kg/d Bortezomib, i.v. (days 0,7). Bortezomib treatment at the higher dose level of 1.3mg/kg could be given on day 0 + 8, but had to be omitted on day 4 and 11 due to median body weight losses of 19% and 20%, respectively. The lower dose of 0.7mg/kg bortezomib could be applied more frequently on day 0, 4 and 11, because this resulted in a median body weight loss of only 6%. Since mice had not fully recovered on day 8, the day 8-treatment was omitted to ensure survival of all animals, resulting in three single, safely administered bortezomib injections on day 0, 4, 11 as depicted in blue. At the end of the observation period (23 days after the first injection), bortezomib-treated, non-tumor bearing mice had reached their initial body weight, almost comparable to untreated controls that achieved a 1.2-fold increase over their initial body weight. (TIF) [file pone.0079939.s006.tif]
