# Supplementary material for: Intratibial Injection of Human Multiple Myeloma Cells in NOD/SCID IL-2Rγ(Null) Mice Mimics Human Myeloma and Serves as a Valuable Tool for the Development of Anticancer Strategies
Source: PLoS One. 2013 Nov 6;8(11):e79939. doi: 10.1371/journal.pone.0079939 (PMC3819303; doi:10.1371/journal.pone.0079939)
Supplement: Table S1 — Survival of immunocompromised mice implanted with L363 or RPMI8226 MMCLs. (DOC) [file pone.0079939.s007.doc]

**Table S1. Survival of immunocompromised mice implanted with L363 or RPMI8226 MMCLs**

| **Mouse strain** | **Cell line** | **Mice (n)** | **Implantation site** | **Median Survival (days)** |
| --- | --- | --- | --- | --- |
| NSG | **L363** | 14 | i.t. | 30 |
| NSG | **L363** | 12 | i.v. | 37 |
| NSG | **L363** | 12 | b.i. | 42 |
|  |  |  |  |  |
| NOD/SCID | **L363** | 12 | i.t. | 49 |
| NOD/SCID | **L363** | 12 | i.v. | 56 |
| NOD/SCID | **L363** | 12 | b.i. | 56 |
|  |  |  |  |  |
| NOD/SCID + CD122 | **L363** | 10 | i.t. | 42 |
| NOD/SCID + CD122 | **L363** | 10 | i.v. | 36 |
| NOD/SCID + CD122 | **L363** | 12 | b.i. | 49 |
|  |  |  |  |  |
|  |  |  |  |  |
| NSG | **RPMI8226** | 12 | i.t. | 35 |
| NSG | **RPMI8226** | 5 | i.v. | >62 |
| NSG | **RPMI8226** | 8 | b.i. | 42 |
|  |  |  |  |  |
| NOD/SCID | **RPMI8226** | 5 | i.t. | >62 |
| NOD/SCID | **RPMI8226** | 5 | i.v. | >62 |
| NOD/SCID | **RPMI8226** | 8 | b.i. | 56 |
|  |  |  |  |  |
| NOD/SCID + CD122 | **RPMI8226** | 8 | b.i. | 58 |

**Abbreviations:**

i.t. : intratibial injection ; i.v.: intravenous injection, b.i.: subcutaneous implantation of juvenile murine long bone

NOD/SCID: NOD.Cg-Prkdc^scid^

NSG: NOD.Cg-Prkdc^scid^Il2rg^tm1^Wjl/SzJ
