# Supplementary material for: Intratibial Injection of Human Multiple Myeloma Cells in NOD/SCID IL-2Rγ(Null) Mice Mimics Human Myeloma and Serves as a Valuable Tool for the Development of Anticancer Strategies
Source: PLoS One. 2013 Nov 6;8(11):e79939. doi: 10.1371/journal.pone.0079939 (PMC3819303; doi:10.1371/journal.pone.0079939)
Supplement: Table S2 — A. Individual MM patient and sample characteristics; B. Summarized clinical MM patient characteristics. (DOC) [file pone.0079939.s008.doc]

**Table S2A. Individual MM patient and sample characteristics**

| **Patient** | **Status** | **prior MM-therapy** | **MM-isotype** | **Sample** | **% PC** |
| --- | --- | --- | --- | --- | --- |
| 1 | relapse | CTD, IEV, ASCT | IgG k | BM | 60 |
| 2 | relapse | MPT, Rd, Cyclo | IgG l | BM | 40 |
| 3 | relapse | ID, IEV, ASCT, allo-SCT, B, CTD, BDD, Rd, RCD | kappa-LC | BM | 50 |
| 4 | relapse | VMP, IEV, ASCT, Benda, CTD, VACOPB | IgG k | BM | 50 |
| 5 | relapse | CD, VACOPB, allo-SCT, DHA | IgM k PCL | PB | 80 |
| 6 | relapse | IEV, ASCT, TD, VCD, Doxo | IgG k | BM | 30 |
| 7 | relapse | TD | IgA k | BM | 20 |
| 8 | relapse | CD | IgG k | BM | 50 |
| 9 | relapse | VCD, IEV, ASCT, Rd | kappa-LC | BM | 90 |
| 10 | relapse | ID, IEV, ASCT, Cyclo, allo-SCT, V, Doxo/D, Benda | IgG l | BM | 20 |
| 11 | relapse | VCD | IgG k | BM | 40 |

**Abbreviations:** CTD: cyclophosphamide. thalidomide, dexamethasone, IEV: ifosphamide, etoposide, epirubicin, ASCT: autologous stem cell transplantation, allo-SCT: allogeneic-SCT, MPT: melphalan, prednisone, bortezomib, Rd: lenalidomide, dexamethasone, cyclo: cyclophosphamide, ID: idarubicine, dexamethasone, BDD: bortezomib, doxorubicin, dexamethasone, RCD: lenalidomide, cyclophosphamide, dexamethasone, VMP: bortezomib, melphalan, prednisone, Benda: bendamustin, VACOPB: doxorubicin, cyclphosphamide, vincristin, bleomycin, etoposide, DAH: cytarabin, TD: thalidomide, dexamethasone, Doxo: doxorubicin, CD: cyclophosphamide, dexamethaone, V: bortezomib

**Table S2B. Summarized clinical MM patient characteristics**

| **MM patient characteristics** | **#** | **Median (range)** |
| --- | --- | --- |
| Number of MM patients | 11 |  |
| Male : female | 7 : 4 |  |
| Age at initial diagnosis (ID) of MM (years) |  | 59 (34-75) |
| MM-type |  |  |
| IgG | 7 |  |
| IgA | 1 |  |
| IgM | 1 |  |
| kappa- : lambda-LC | 9 : 2 |  |
| MM-stage according to |  |  |
| Salmon & Durie | IIA: 1, IIIA:10 |  |
| ISS | I:3, II:5, III:3 |  |
| Disease status (relapse) | 11 |  |
| BM-infiltration (% PC) |  | 50 (20-90) |
| Cytogenetic risk according to Fonseca | SR:7, HR:4 |  |
| Prior therapy lines (range) |  | 2 (1-7) |
| Standard vs. high-dose therapy (ASCT or allo-SCT) | 4 : 7 |  |
| Alive vs. deceased | 4 : 7 |  |
| MM duration (from ID to death or last follow-up* (months) |  | 62 (1-100) |

**Abbreviations:** LC: light-chains, BM: bone marrow, ID: initial diagnosis, PC: plasma cells, *last follow-up: 7/2013
